# Structure, Ecotoxicity, Redox and Bactericidal Activity of Cu-Containing Nanocrystalline Ferrites

**DOI:** 10.3390/molecules30224454

**Published:** 2025-11-19

**Authors:** Todor R. Karadimov, Elena P. Nenova, Elitsa L. Pavlova, Iliana A. Ivanova, Milena T. Georgieva, Peter A. Georgiev

**Affiliations:** 1 Department of Condensed Matter Physics and Microelectronics, Faculty of Physics, Sofia University “St. Kliment Ohridski”, 1164 Sofia, Bulgaria; tkaradimov@uni-sofia.bg (T.R.K.); mgeorgieva@phys.uni-sofia.bg (M.T.G.); 2 Faculty of Biology, Sofia University “St. Kliment Ohridski”, 8 Dragan Tsankov Blvd, 1164 Sofia, Bulgaria; nenova@uni-sofia.bg (E.P.N.); iaivanova@biofac.uni-sofia.bg (I.A.I.); 3 Optics and Spectroscopy Department, Faculty of Physics, Sofia University “St. Kliment Ohridski”, 1164 Sofia, Bulgaria; elli_pavlova@abv.bg

**Keywords:** nanocrystalline ceramics, superparamagnetism, metallic copper particles, antimicrobial activity, ROS, ecotoxicity

## Abstract

Cu-modified ferrites, prepared by solvothermal syntheses, at up to 200 °C, show the presence of copper metal particles, embedded in ferrite nanocrystalline particle agglomerates. Notably, these metallic copper micron-sized crystallites were dramatically reduced in size, down to a few tens of nanometers, when part of the copper dopant was replaced by zinc. All materials were magnetic due to the presence of the cubic spinel phase, being ferrimagnetic, measured with external fields up to 6000 Oe, showing a narrow hysteresis of 89 Oe for the largest particle size copper ferrite material of 15 nm. Superparamagnetic behavior was observed for the smallest size, e.g., 11 nm, Cu-doped and the zinc-doped, 9–10 nm average particle size ferrites. The redox activity of the materials was studied in free-radical oxidation reactions (pH 7.4, physiological and pH 8.5, optimal) by the chemiluminescent method with (i) Fenton’s reagent (^·^OH, ^·^OOH); (ii) H_2_O_2_; and (iii) O_2_^·−^ radicals. All materials presented extremely strong inhibitory activities (converted to prooxidant only at pH 7.4 in system iii, excluding the largest isolated copper-particle-containing material, which remained inhibitory). The materials’ antimicrobial potential was checked on Gram-positive and Gram-negative bacteria, *Escherichia coli* ATCC 25922, and *Staphylococcus aureus* ATCC 25923 via two classical methods, namely the spot and well diffusion tests in agar medium. The above tests included a nanocrystalline CuO, tenorite, as a reference material too. The *Daphnia magna* ecotoxicity test showed that all of the investigated materials are rather toxic, and since daphnia is a key component in freshwater ecosystems, the toxicity even at low concentrations may have significant consequences for the ecological balance. This requires careful monitoring and assessment of the possible use or disposal of these nanomaterials in the environment.

## 1. Introduction

At the lower limits of spatial atomic order, nanocrystalline materials incorporating bioactive elements in atomic/ionic forms and magnetic components or phase-separated surface-deposited inclusions offer a large and diverse framework of multifunctional materials. Considering mainly inorganic materials, these include metallic nanoparticles and simple and complex metal oxides. Properties originating from the reduced particle sizes, e.g., tissue/cell permeabilities and facile body release, relatively large specific surface area, and abundance of active surface sites, are often combined with magnetism—ferro-/ferri- or superparamagnetism and biocompatibility to establish novel key applications in biomedicine [1,2,3]. These include hyperthermia and drug delivery [4], anticancer drug delivery agents [5], and direct cancer annihilation by multifunctional nanomaterials [6]. To complement the range of intensively studied applications, antimicrobial action and wound healing [7,8,9,10], drinking and industrial water disinfection and purification [11,12,13,14,15], and radioactive species sequestration [16,17,18] should be noted as key points in the security and sustainability of modern clean environments. Furthermore, the multifunctionality of many nanomaterials underpins their added value as “theranostics”, where one property ensures their diagnostic capability while another ensures their therapeutic one [19,20,21]. The growing worldwide concern for antibiotic microbial resistance, and on the other hand, antibiotic environmental pollution, food and drinking water contamination by existing and new pathogens, are already identified as the cause of death for nearly a million people per year with a gloomy perspective to increase dramatically in the forthcoming years [22,23,24]. Thus, there are prominent and constant driving forces stimulating research on the discovery and development of novel environmentally friendly materials to replace/complement antibiotics, combat new pathogens, and generally ensure a clean and safe environment.

Known for centuries and one of the most explored bioactive species, particularly for its bactericidal action, is silver [25,26]. Its active form may be of a different type: ionic, charged, and neutral cluster species as well as nanoparticles, carried on surfaces and in micropores [27]. We have studied the antimicrobial activity and toxicity of various silver species dispersed in zeolites [28] and found that high content, e.g., >30% by weight, silver nanoparticle-loaded zeolites possessed very high antimicrobial activity, while an order of magnitude lower content of Ag^+^ exchanged zeolite showed a comparable bactericidal effect. Both materials, namely Ag-Xcl(Na-X type zeolite) and SA25-Ag (ZSM-25 zeolite with Si-to-Al = 25), appeared to be highly toxic to *Daphnia magna* [28]. While all materials, carrying predominantly either ionic silver species or nanoparticles, showed decreased activity with the time of storage under ambient conditions, a clear conclusion on which exactly was the active species was not possible to draw. A scenario in which silver nanoparticles could also release more active ionic or cluster silver species at certain stages could not be excluded [28]. However, it is clear that a careful and accurate material characterization, with suitable experimental techniques, is essential for the successful development of these materials [29], taking into account that some species and materials as a whole may undergo structural changes due to the influence of the characterization probe, as observed in our case with silver reduction during X-ray analyses [28].

Zinc, with its ionic species mostly carried as a component in ZnO nanoparticles, is very popular due to its practical biocompatibility, and it has already found real-life applications [30]. Despite the vast number of research works and research techniques employed, the exact mechanisms involved in the antibacterial action, in which cell growth is either terminated or inhibited, or tumor cell annihilation is observed, are not quite clear as it is often not clear what is the exact nature of the active species—ionic, clusters, or nanoparticles. Various antibacterial action mechanisms have been considered particularly for zinc oxide nanoparticles [30] that may be generalized for all nanoparticles, not excluding other possible mechanisms too. Although still debatable, the accepted main mechanisms of antibacterial activity include (i) reactive oxygen species (ROS) generation [31], induced by ionic species in a zeolite carrier [32] entering bacterial cells. The role of singlet oxygen generation by nanoparticles has been noted in some studies [33,34] too. (ii) The release of ionic species on their own or in conjunction with ROS action has been considered [35]. Notably, the authors of the latter study [35] found that the bactericidal activity was rather directly related to the release of ionic species, Zn^2+^, whereas the generation of ROS was not. (iii) Nanoparticle penetration through cell membranes and inhibition of cell metabolism [36] or direct, i.e., mechanical cell membrane damage and leakage, have also been noted [37]. In other cases, it has been found that metallic nanoparticles may cause cell DNA damage [38]. Due to a holistic structure–activity approach and using a large experimental results database [39], metallic nanoparticles, including Co, Ni, Cu, Zn, Ag, Au, and Pd, have been classified as toxic, while W, Sn, and Al nanoparticles have been classified as nontoxic [39]. The same study concluded that metal oxide nanoparticles of Ag_2_O, Co_2_O_3_, CoO, CuO, Cu_2_O, Mn_2_O_3_, Mn_3_O_4_, Er_2_O_3_, Ni_2_O_3_, Yb_2_O_3_, and ZnO are toxic. On the other hand, Al_2_O_3_, CaO, CeO_2_, Fe_2_O_3_, Ga_2_O_3_, Gd_2_O_3_, HfO_2_, MgO, MoO_3_, SiO_2_, SnO_2_, TiO_2_, WO_3_, Y_2_O_3_, and ZrO_2_ appear nontoxic. For medicinal and related applications, however, it is the active species concentration window that needs to be adequately determined. This would allow for the use of a particular nanomaterial at concentrations that are harmless for the host but detrimental for the targeted pathogens, and especially drug-resistant strains. Henceforth, intensive research efforts have been devoted to studying the biocompatibility of Fe_3_O_4_, MnFe_2_O_4_, ZnFe_2_O_4_, and other spinel structure nanosized materials with more than direct antibacterial properties. Others include controlled-release drug carriers under pH activation, magnetic resonance imaging (MRI) enhancement agents [40,41], and tumor cell annihilation via hyperthermia, where an external alternative magnetic field is applied on the particles, causing local heating [42,43].

Iron oxide exists in three different forms, namely cubic spinel Fe_3_O_4_ and space group Fd3m, known as the mineral magnetite, containing both di- and three valent iron species. Magnetite may be oxidized to form maghemite, γ-F_2_O_3_, which is a defect structure of magnetite, containing only trivalent iron in the same crystal lattice. At elevated temperatures, maghemite is converted to hematite α-F_2_O_3_ with hexagonal lattice and space group R$\bar{3}$c. Both magnetite and maghemite are ferrimagnetic, with the latter being a defect structure of the other, with some vacant iron sites, due to the higher oxidation state.

**Figure 1:**
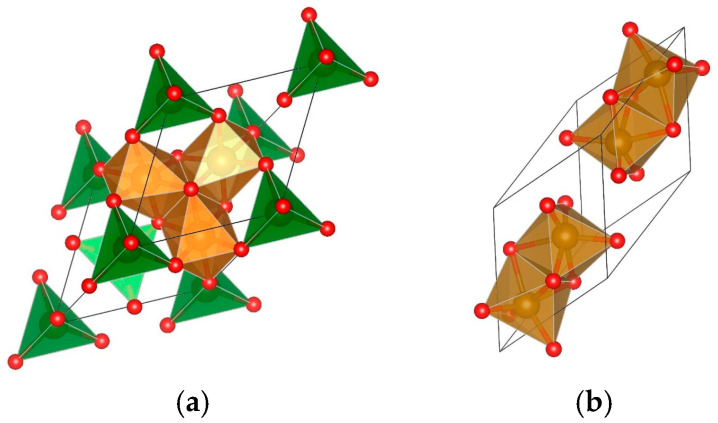
Primitive unit cell of magnetite in (**a**), and the same for hematite in (**b**). Oxygen atoms, forming the tetrahedral and octahedral sites, as explained in the text, are presented in red.

Magnetite is perhaps the most studied iron oxide form for biomedical purposes, but there are also many other applications related to its magnetic nature. The reason for this may be found in its flexible crystal structure, which is able to accommodate various other metal ions in the place of some of the iron ions. This, in turn, provides a broader framework for the creation of materials with variable physicochemical properties, including bioactivity-related ones. The corresponding crystal structure of magnetite may be also written as FeFe_2_O_4_, namely the cubic spinel with a general formula AB_2_O_4_, and in its primitive cell representation, shown in Figure 1a, it consists of two structural building blocks: (i) two tetrahedra form site A, i.e., AO_4_^−6^ units, and (ii) the voids in between the tetrahedra make four octahedral sites where the charge compensating B ions resides. Hematite, the thermodynamically most stable, nonmagnetic phase, shown in Figure 1b, consists of only octahedra. The spinel is called “normal”, when all divalent ions reside in the A-sites and all three valent ions reside in the B-sites, as is the case for ZnFe_2_O_4_ [44,45]. The spinel is called inverse, as is the magnetite, when half of the three valent iron ions are positioned in the tetrahedral sites, while the divalent ions occupy half of the octahedral B-sites. Such inversed spinel is the nickel ferrite, while manganese ferrite is only partially inverse, and in thermodynamic equilibrium under normal conditions, 80% of the divalent ions reside in the A-sites [44,45]. However, in nanocrystalline versions of these materials, including other metal ion substitutes like Co, Cr, Cu, Ca, and Li, various degrees of inversion are often observed.

In the present work, we have focused on copper-substituted ferrites which, due to the well-known antimicrobial and generally toxic properties of Cu [46,47], CuO [48,49], Cu_2_O [50,51], and other copper-containing compounds with biological and medicinal functionalities [52,53], have the potential to combine the aforementioned biological properties with magnetism at the nanocrystalline level. Copper specifically is very important to study because of its possible crucial role in the balance of metals in the brain, affecting the development of Alzheimer’s disease, dementia, and Parkinson’s disease [47]. In its equilibrium bulk state, at temperatures below 427 °C, Cu-ferrite crystalizes in tetragonal unit cell, space group I4_1_/amd. Above this temperature, the compound adopts the cubic spinel structure [53,54]. The phase compositions and the tetragonal-to-cubic phase transition temperature have been studied in a number of studies as a function of particle size and synthesis routes, showing no definitive correlation between crystallite size and phase composition nor transition temperature [55,56,57,58,59]. Attempts to prepare the cubic spinel phase at near room temperature have led to obtaining two-phase samples or even expelling part of the copper in tenorite extra phase [58,59]. The potential biological applications of copper ferrite, and particularly the cubic spinel phase, have already attracted research interests. It has been found, for instance, that increasing the copper content in cobalt ferrite nanoparticles substantially enhances their antimicrobial activity [60]. While the authors of this study assumed pure cubic spinel phase materials for all anticipated stoichiometries, in their diffraction patterns, the presence of extra reflections can be seen. These were identified as due to CuO, tenorite, phase as found in another study on the synthesis of copper ferrites at different pH values [61]. Notably, the amount of the extra CuO phase increased upon changing the synthesis pH from 12 down to 8 [61]. Thus, a hypothesis in which the active component is actually copper monoxide, tenorite [48,61], with an increasing amount that correlates well with the enhancement of the observed antimicrobial activity [61] seems reasonable. The stable tetragonal form of copper ferrite, on the other hand, has been tested alongside zinc ferrite, showing high antioxidant and antibacterial efficacy [62], further promoting the possible bio- and medicinal applications of copper-dopped ferrites. Remarkably strong bactericidal action has been observed for Cu(I) species formed at the surface of copper ferrite nanoparticles via partial reduction by hydroxylamine (NH_2_OH) treatment [63]. Regardless of the exact crystallographic phase, but rather due to the electronic state of the active, in this case, ionic surface species were able to generate ROS at high constant rates inside microbial cell bodies [61]. Quite often, at the scales of a few nanometer particles and when biological functionalities are targeted [64,65], including the activation of key functions under near-infrared irradiation [66], the cubic spinel polymorph of copper ferrite is assumed and has seemingly been observed, usually by PXRD, to be the dominating and consequently active material form. To elucidate further the role of the material crystalline state and order, we have focused on the microstructural and magnetic properties of our copper-containing ferrite materials as key factors responsible for biologically important functionalities such as antimicrobial action, redox activity, and ecotoxicity. As mentioned earlier [31], special attention was paid to the generation of ROS and prooxidant activity as an important biomarker of the antibacterial activity and the oxidation level inside of the organism. On the contrary, low levels of these reactions and products are usually demonstrative for antioxidant (inhibitory) effects [67,68].

## 2. Results and Discussion

**Figure 2:**
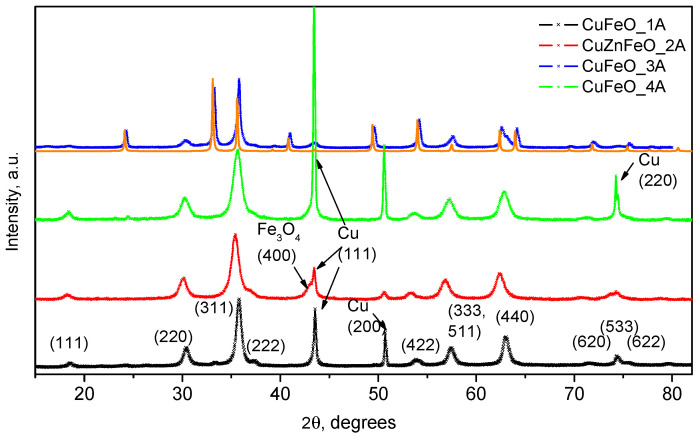
PXRD patterns of the four sample materials, as indicated in the figure legend. The solid orange line, underneath the top pattern of CuFeO_3A, is due to a calculated PXRD profile of hematite.

XRPD data of the dry powder materials are shown in Figure 2 over most of the observable diffraction intensity for these structures. As seen in the same figure, for samples CuFeO_1A, CuZnFeO_2A, and CuFeO_4A, the scattered integral intensity is dominated by the magnetite cubic spinel phase, as indexed in the diffractogram of CuFeO_1A, in Figure 2. Sharp extra peaks of the secondary phase are identified as metallic copper (SG. Fm3m, 224), showing the presence of the most intense reflections (111), (200), and (220), Figure 2. The diffractogram of the CuFeO_3A material is dominated by the most stable α-Fe_2_O_3_ phase, namely hematite, hexagonal space group Rc (No. 167). This is shown alongside a computed diffraction pattern for better identification of the measured reflexes since there is some coincidence with those of the cubic spinel phase. This sample contains significant amounts of the cubic spinel phase too, as indicated by the presence of the (220), (311), (333), (511), and (440) reflexes. Notably, no metallic copper is observed in the PXRD profile of this sample.

On the other hand, the lattice parameters of the cubic spinel phases in samples CuFeO_1A and CuFeO_4A are very close to those of pure magnetite Fe_3_O_4_ [69,70,71], while that of CuZnFeO_2A agrees quite well with the lattice parameter of nanocrystalline ZnFe_2_O_4_ [45,72]. Better insight at the diffraction patterns, around the metallic copper phase reflections, is provided in the Supplementary Material, Figure S1. An example profile fitting to extract the constituent phases crystal lattice parameters is shown in Figure S2. The main microstructural and room-temperature magnetic properties are summarized in Table 1.

**Figure 3:**
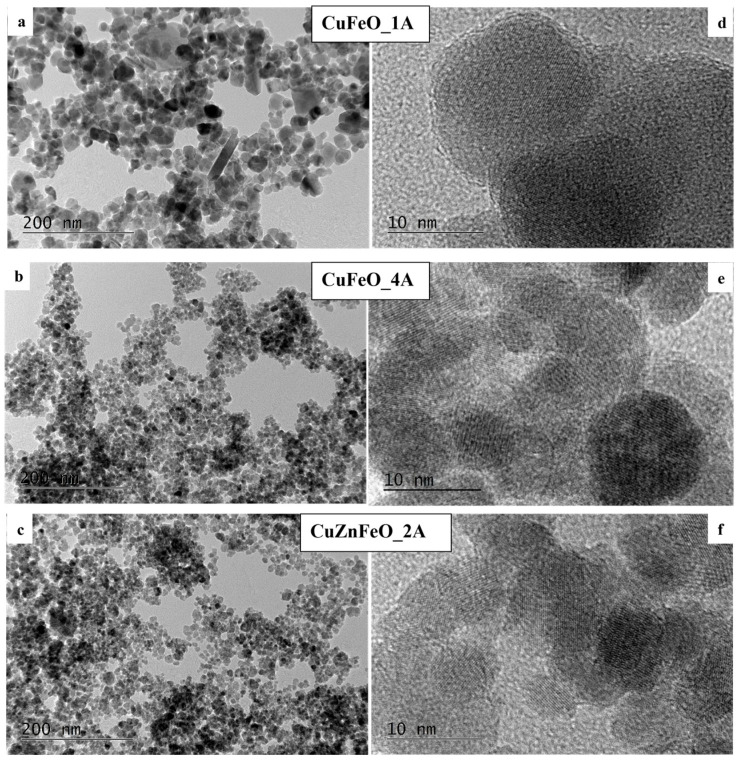
TEM images of the CuFeO_1A—(**a**), CuFeO_4A—(**b**), and CuZnFeO_2A—(**c**). HRTEMs of the same samples are shown in (**d**–**f**) to observe the typical crystallite particle sizes.

**Figure 4:**
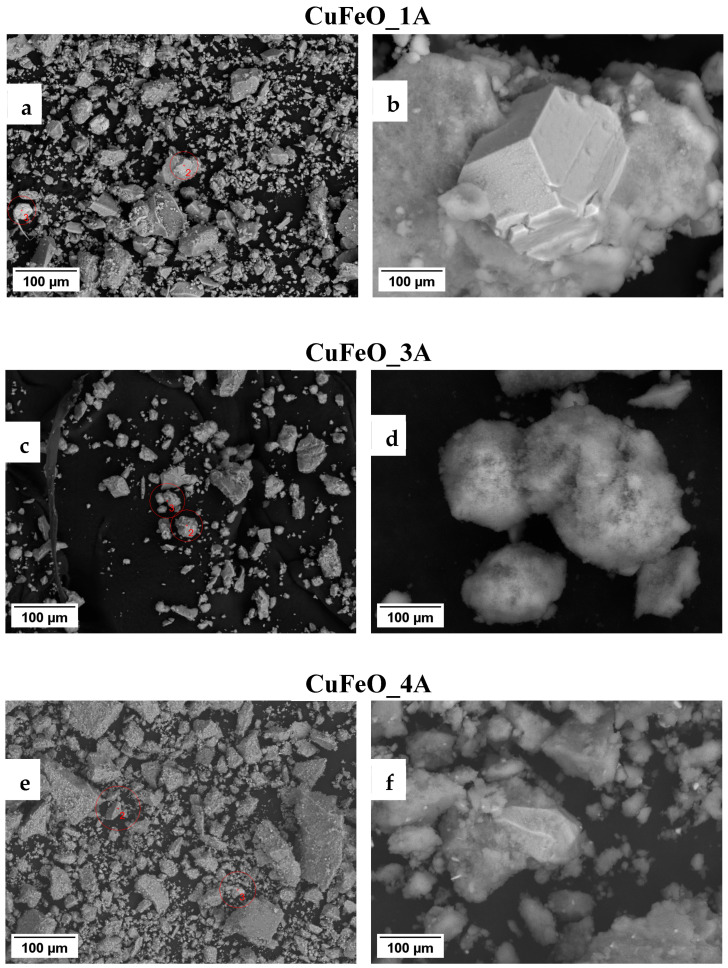
SEM images of the CuFeO_1A—(**a**,**b**), _3A—(**c**,**d**), and _4A—(**e**,**f**) samples, illustrating the agglomerated powder morphology—left side—and isolated copper crystalline particles—on the right side. Accelerating voltage was 20 kV, at SEM WD ~9 ÷ 9.5 mm, magnifications ×500 and ×5000, accordingly.

These observations suggest that only very small amounts of copper may have entered the spinel crystal structures, and most of the copper has precipitated in the form of metallic particles. The estimated X-ray particle sizes of the cubic spinel ferrite phases agree well with the high-resolution transmission electron microscopy (HRTEM) observations. Notably, the observed PXRD reflections of the cubic copper phase in CuFeO_1A and CuFeO_4A samples contain no sample line broadening. On the contrary, and although following very similar synthetic procedure as for CuFeO_4A, in the CuZnFeO_2A material, the copper phase reflections are significantly broadened, comparable to the host spinel phases, as seen in Figure 2. Clearly, the metallic phase in this sample is nanocrystalline too, with an average crystallite size of about 25–30 nm, as derived from the corresponding diffraction line broadening. HRTEM images for three of our samples are shown in conventional—Figure 3a–c—and in high-resolution mode—Figure 3d–f. The macroscopic morphologies of the samples are visualized in the first set, evidently showing a relatively uniform size distribution. Sample CuFeO_1A contains larger nanoparticles with average sizes in the range of 15–20 nm, whereas samples CuFeO_4A and CuZnFeO_2A consist of nanoparticles with average diameters of 5–10 nm. In the high-resolution mode TEM images, Figure 3d–f, the lattice planes can be seen clearly for each visible grain/particle, which proves that all samples are nanocrystalline, even those with particle sizes down to 5 nm. Scanning electron microscopy (SEM) images of only the Cu-containing samples were collected, searching for metallic copper particles (no such particles were expected in CuZnFeO_2A due to the PXRD results, Figure 2), and are shown in Figure 4. These elucidate the presence of copper metal particles in the form of fine, well-shaped micron-sized crystallites in samples CuFeO_1A and CuFeO_4A. Details of the corresponding EDS analyses and element distribution mapping are provided in the Supplementary Information.

On the other hand, no well-defined copper metal particles are seen in the SEM images of the CuFeO_3A material, with the presence of the copper spectrum spreading on parts of the sample, as seen in the corresponding element map to be more like an irregular surface coating. We recall that all powder materials were collected from their mother liquors and out from the washing solvent using a magnet. However, clearly not only magnetic phases present in the collected powders, and upon visual inspection, no powder material had remained in the corresponding liquid solutions. Hence, the magnetic and nonmagnetic phases must be incorporated into one another. Furthermore, all four materials appear dark brown to black, while pure hematite is orange-reddish.

Copper-substituted hematite materials have been studied before [73,74,75,76], showing that its hexagonal phase takes up to 5–6% wt Cu(II) [74,75,76] and above metallic copper is precipitated [74]. The determined lattice parameters in our hematite phase, on the other hand, agree quite well with those of pure hematite [74,76], and we might then again assume that the CuFeO_3A material is a composite of hematite, magnetite, and nanostructured copper and/or copper oxide phases. Note that if the copper oxide phase consists of very small, a few nanometers, crystallite, and not a very high amount, this would preclude its clear observation in the PXRD pattern. The antibacterial activity of Cu-doped hematites, in particular, clearly increases with the increase in the copper content [74], further confirming that most likely released Cu-ionic species are the active antimicrobial component.

### 2.1. Magnetic Properties

**Figure 5:**
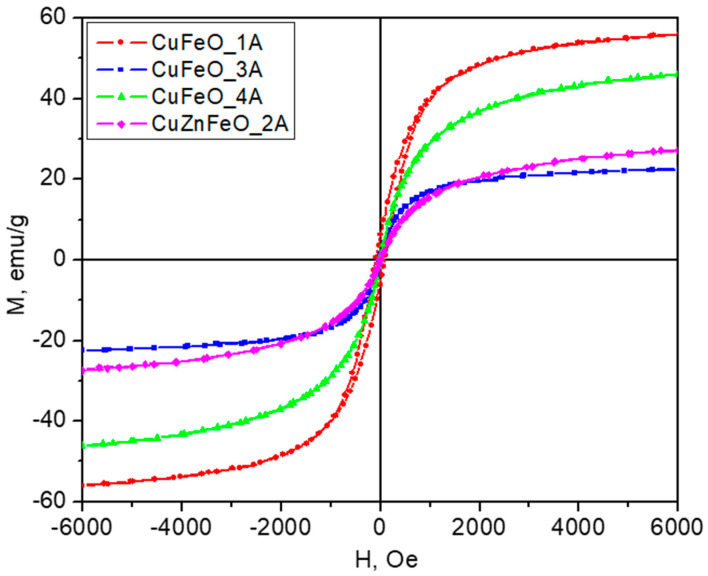
Magnetic hysteresis loops measured at room temperature. The inset shows the region of small magnetic fields to visualize the hysteresis effects.

All samples show magnetic properties at room temperatures, as summarized in Table 1. The corresponding magnetization curves at ambient conditions and for external magnetic fields up to 6000 Oe are displayed in Figure 5. Sample CuFeO_1A has the highest maximal magnetization of 56 emu/g (at 6 kOe—the maximum field intensity available on the instrument) and the largest coercivity. Its behavior is ferrimagnetic, typical for the ferrite materials. On the other hand, sample CuFeO_4A has practically zero coercivity and is therefore superparamagnetic, attaining a maximal magnetization of 46 emu/g at 6 kOe. The observed relatively high magnetization is most likely due to the good crystallinity of the nanoparticles seen on the HRTEM images too, as shown in Figure 3. These magnetic properties—high magnetic moment and zero coercivity—are quite suitable for biomedical applications such as drug deliveries. One can see from the TEM images that sample CuFeO_1A consists of larger particles above 15 nm in diameter, whereas sample CuFeO_4A consists of particles less than 10 nm in diameter. Particles less than 10nm are very likely to be superparamagnetic, which explains the difference in the magnetic properties of the abovementioned samples.

The CuFeO_3A material is ferrimagnetic at room temperature, with a maximal magnetization much lower than that for CuFeO_1A, by about a factor of three and a factor of two lower than that of CuFeO_4A, indicating that the magnetic phase quantity in this material must have been reduced by the corresponding amount, which generally agrees with the PXRD observation. This is mainly due to the predominant existence of the antiferromagnetic phase at room temperature [77] and the hematite phase in this sample, thus reducing the overall net magnetic moment. Note that the antiferromagnetic state shows only weak paramagnetism in external magnetic fields and hence contributes a negligible amount to the magnetization of the material, only at high external field intensity values. The addition of Zn in the copper ferrite structure, sample CuZnFeO_2A, results in purely superparamagnetic properties at room temperature, Figure 5, presumably due to the small size of the nanoparticles, mostly less than 10 nm, as seen in the TEM images, Figure 3. It is worth noting that since no extra zinc-containing phases are detected, the spinel phase must be a Zn-substituted ferrite, e.g., Zn_x_Fe_1−x_Fe_2_O_4_ which is known as being ferrimagnetic at ambient temperatures, in contrast to the stoichiometric normal spinel ZnFe_2_O_4_ which would be paramagnetic above 10 K [44,45]. However, at the nanoscale, and when prepared at relatively low temperatures, some inversion degree in ZnFe_2_O_4_ may exist too, converting the material into a ferrimagnet at room temperatures [45]. Since this material additionally contains diamagnetic copper metal phase, Figure 2, a corresponding reduction in the expected magnetization should be expected.

### 2.2. Redox Activities

To study the effect of the newly synthesized materials on the kinetics of free-radical oxidation reactions, we applied the activated chemiluminescent method using the probe lucigenin in three model systems [78]. Usually medium with higher alkalinity favors radical generation and enables the achievement of reliable measurements and comparable differences. Two different pH levels were tested, pH 7.4 and pH 8.5, physiological and alkaline (favoring ROS generation). Three ex vivo model systems were implemented in buffer solutions [78]. As for the other biological studies below, an own nanocrystalline CuO (nCuO) material was tested along with the Cu-ferrite materials as an informal reference. Its structural characteristics, by means of PXRD and TEM data, are provided in the Supplementary Information file Figure S3.

#### 2.2.1. System I

The Fenton’s reaction between Fe^2+^ ions and H_2_O_2_ produces highly reactive, short-living radicals. Usually, the achieved chemiluminescent emission is much higher than the one from other mixtures:

(1) Fe^2+^ + H_2_O_2_ → Fe^3+^ + ^·^OH + ^−^OH
(2) Fe^3+^ + H_2_O_2_ → Fe^2+^ + ^·^OOH + H^+^

**Figure 6:**
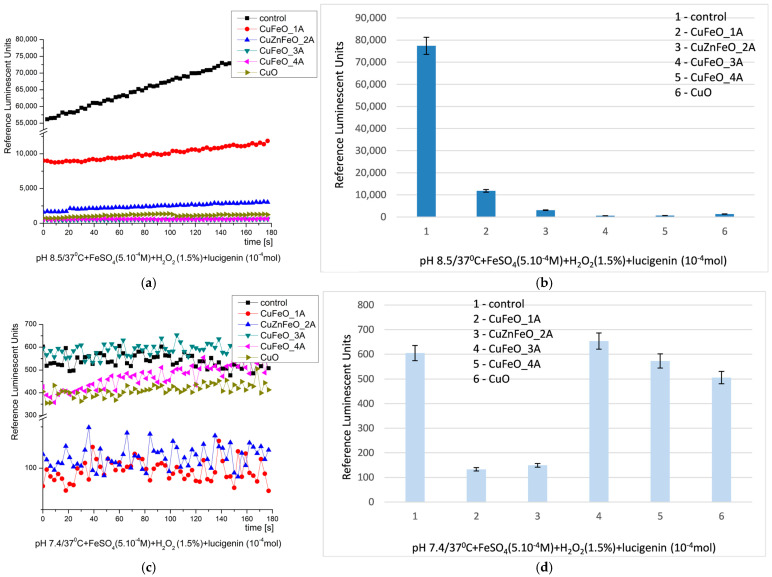
Fenton’s chemiluminescence induced by ^·^OH and ^·^OOH radicals at pH 8.5 ((**a**) kinetics, (**b**) maximums) and pH 7.4 ((**c**) kinetics, (**d**) maximums) and the effect of newly synthesized nano-ceramic materials (*p* ≤ 0.05).

At pH 8.5, all the observed reactions, except the control, possessed similar kinetics—it was like a plateau, slightly increasing with time. That was indicative for the ROS taking part in the reaction and being released in the solution with time. The control reaction emitted the highest signal (100%), maximum ~77,340 RLU. All the tested materials presented definitive suppression and strong inhibitory activity against the specifically generated ROS. CuFeO_1A showed lowest inhibition—6.5 times; CuZnFeO_2A presented a stronger inhibition—~25 times; CuFeO_3A and CuFeO_4A suppressed the signal 128 and 113 folds; and the signal detected from nCuO was 56 times lower than the control, Figure 6 [28,68,78].

At pH 7.4 (physiological), the kinetic curves were not smooth and had various fluctuations. But all the tested materials suppressed the chemiluminescence signal and also presented inhibition: CuFeO_1A—4.5 fold, CuZnFeO_2A—4.2 fold, CuFeO_4A, and nCuO—5% and 16% inhibition, respectively; CuFeO_3A had no effect, Figure 6. When the signal is suppressed, this is indicative of the neutralization of the present ROS in the system, and hence indicative of the antioxidant/inhibitory properties of the material. On the contrary, in the reactions where the signal is higher than the control, the material is oozing metal ions or other reagents, forming ROS or others, taking part in the chosen reaction.

#### 2.2.2. System II

**Figure 7:**
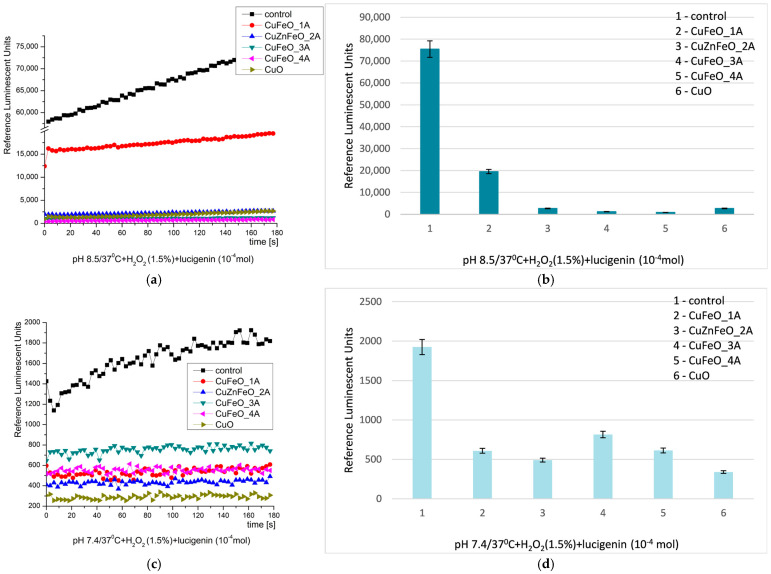
H_2_O_2_ chemiluminescence induced by H_2_O_2_ at pH 8.5 ((**a**) kinetics, (**b**) maximums) and pH 7.4 ((**c**) kinetics, (**d**) maximums) and the effect of newly synthesized nano-ceramic materials (*p* ≤ 0.05).

In this system, the strong oxidant hydrogen peroxide serves both as an oxidizing agent and an ROS. At alkaline pH, the kinetics curves were smooth and the signal in all the reactions was increasing slightly in time. The control system emitted most light. All the other tested samples demonstrated strong inhibition on the reaction: CuFeO_1A—almost 4 times, CuZnFeO_2A—28 times, CuFeO_3A—almost 63 times, CuFeO_4A—88 times (highest inhibition), and nCuO—28 times (similar to CuZnFeO_2A), Figure 7 [28,68,78]. This is indicative of all the tested materials efficiently suppressing oxidation by H_2_O_2_ at alkaline conditions.

At pH 7.4, all the tested materials confirmed their inhibitory properties against H_2_O_2_. CuFeO_1A, CuZnFeO_2A, and CuFeO_4A reduced the chemiluminescent signal three times, CuFeO_3A reduced it twice, and nCuO reduced it almost six times. The kinetic curves presented fluctuating plateaus, Figure 7. H_2_O_2_ always acts as a strong oxidizing agent, even at very low concentrations.

The described antioxidizing H_2_O_2_ activity of the tested materials, confirmed by the above results, suggests that they can effectively scavenge or decompose this ROS under physiological conditions too. This behaviour implies redox-modulating properties that could be beneficial in reducing oxidative stress in biological environments. Such inhibition of H_2_O_2_-induced oxidation indicates that these metal oxide composites may act as efficient antioxidant mimetics, possibly through surface redox reactions.

#### 2.2.3. System III

The O_2_^·−^ generation in this system is following the chemical scheme of Nishikimi et al. [79,80]:

PhMS + NAD.H + H^+^ ⟶ PhMS.H_2_ + NAD^+^ (1)

PhMS.H_2_ + PhMS ⟶ 2PhMS.H^·^ (2)

PhMS.H^·^ + O_2_ ⟶ PhMS + O_2_^·−^ + H^+^ (3)

The superoxide radical is extremely short living and very aggressive.

**Figure 8:**
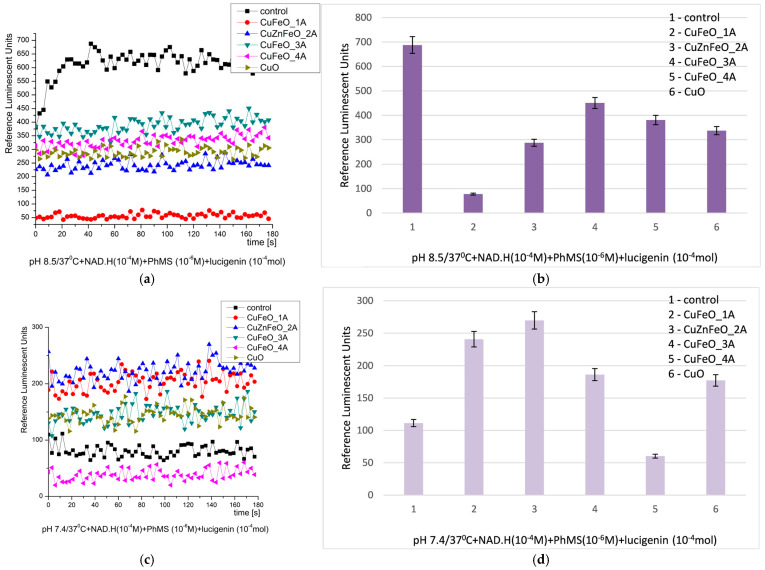
NAD.H-PhMS chemiluminescence induced by O_2_^·−^ radicals at pH 8.5 ((**a**) kinetics, (**b**) maximums) and pH 7.4 ((**c**) kinetics, (**d**) maximums) and the effect of newly synthesized nano-ceramic materials (*p* ≤ 0.05).

At pH 8.5, all the tested materials demonstrated once more inhibitory properties and effective neutralization against the generated ROS. Their inhibition activity was as follows: CuFeO_1A—8.8 times; all the other materials presented close effects—less or more than 2 folds inhibition of the reaction. The kinetics curves were fluctuating in a plateau shape, Figure 8 [28,68,79].

At pH 7.4 (physiological), the observed inhibitory activity was converted into prooxidant. That effect was observed only in that oxidation model system and conditions. The prooxidant effects varied between 1.6 and 2.4 times with respect to the control in the sequence CuFeO_1A, CuZnFeO_2A, CuFeO_3A, and nCuO. Inhibitory properties in those conditions possessed only CuFeO_4A, decreasing the chemiluminescent signal to less than two times with respect to the control. The kinetic curves were fluctuating in a plateau shape too, Figure 8. The effects observed for all tested materials were stable in time.

In comparison to the control reactions, the efficiency of the investigated materials can be summarized as follows, Table 2:

All the observed effects demonstrate that most of these materials do not provoke the accumulation of ROS and would not burden the physiological processes that are connected with their generation, breaking the extremely sensitive oxidation equilibrium in the body. Most of them exhibited very well-defined inhibitory effects against the already generated ROS, which was stable in time. All the tested nano-ceramic materials showed clear inhibitory/antioxidant activity, especially at alkaline pH (8.5), effectively suppressing ROS generation and oxidation reactions. The inhibition was pH-dependent, being stronger under alkaline conditions than at physiological pH (7.4). Overall, the materials did not promote ROS accumulation and maintained redox balance, indicating good physiological compatibility and stable, time-persistent effects suitable for redox-modulating applications.

### 2.3. Antimicrobial Activities

The results from the antimicrobial tests, summarized in Table 3 and Table 4, evidence similar activity of the copper-containing ferrite nanoparticles for both Gram-positive and Gram-negative bacteria. The nanocrystalline tenorite powder (nCuO), used as a reference, was by a factor of 10 more toxic than the composite ferrite—Minimal Bactericidal Concentration (MBC) on both bacteria (Table 3 and Table 4). *S. aureus* ATCC25923 was somewhat more resistant as CuFeO_1A and CuFeO_3A had no effect on it, in concentrations of 10 mg/mL, in contrast to *E. coli* ATCC25923. The other two materials, CuFeO_4A and CuZnFeO_2A, had a similar effect on both bacteria: with Minimal Bactericidal Concentration (MBC) of 10 mg/mL and Minimal Inhibition Concentration (MIC) of 5 mg/mL. These results suggest higher biocompatibility of CuFeO_1A and CuFeO_3A, and a lower one for CuZnFeO_2A and CuFeO_4A. Our present results may be directly compared to previous studies with the same system [67], in which the authors also observed higher sensitivity of *E. coli* in the presence of the copper-containing ferrites than *S. aureus.* Notably, the copper ferrite materials in that earlier study [67] showed very much improved antibacterial efficiency when irradiated by monochromatic IR light (808 nm), which was able to absorb and convert that extraordinarily efficiently, as high as nearly 29% [66], which is quite surprising for a semiconductor with a band gap of around 2 eV [81,82]. A closer inspection of the PXRD data presented in Figure 2g in Ref. [66] reveals the presence of very sharp, high-intensity lines on top of the (400), (200), and (533) spinel reflexes, much sharper than the (220), (311), (511), and (440) lines, which are clearly very much broadened due to the nanocrystalline character of that material [66]. The sharp intense lines in that study too coincide with the (111), (200), and (220) lines of metallic copper, and, in fact, the corresponding diffractogram is very similar to that of our CuFeO_4A material, Figure 2, in the present study.

The presence of extra-phase metallic copper in [66], on the other hand, could mean that the nanocrystalline majority phase is actually Fe_3_O_4_, magnetite, or the same with little copper substitution, in which case the band gap would drop down to 0.1–0.3 eV [83], making the material a very good absorber in the IR range. This would mean, however, that those authors actually investigated some magnetite Fe_3_O_4_ (explaining also the high magnetization values) along with the presence of large metallic copper crystallites [67] and the extraordinary IR light to heat conversion efficiency (and the corresponding bactericidal efficiency under IR) that may be due to a cooperative effect between the magnetite nanocrystalline material and metallic copper, which is a plasmonic metal too. However, in our studies, all of the copper-containing materials on their own, i.e., without external assistance, behave as rather weak bactericidal agents.

### 2.4. Ecotoxicity

**Figure 9:**
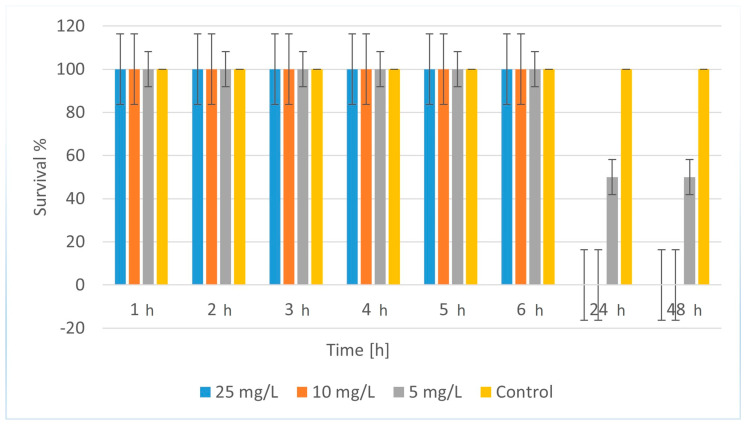
Survival of *Daphnia magna* treated with CuFeO_1A.

The results of the experiments with CuFeO_1A, Figure 9, showed that at high concentrations (25 and 10 mg/L), daphnia was alive during the first 6 h (100% survival). After 24 h, survival dropped sharply to 0% and remained so at the 48^th^ h. This indicates strong toxicity with a rapid lethal effect that occurred after the 6^th^ h. At 5 mg/L, daphnia remained 100% alive during the first 6 h. After 24 h, survival was 50% and remained at this level at the 48^th^ h. This result indicates moderate toxicity at this concentration. That is, CuFeO_1A exhibited pronounced toxicity on daphnia, with mortality occurring mainly after 6 h and increasing up to 24 h. The toxic effect was strongly concentration-dependent, with complete mortality at 10 and 25 mg/L and partial at 5 mg/L. The control showed that the toxicity was due to the substance, not the conditions. An LC_50_ for CuFeO_1A was calculated based on the data obtained, for 24 and 48 h (since there was significant mortality there). Here, 50% mortality was exactly at 5 mg/L, so LC_50_ (24 and 48 h) ≈ 5 mg/L. The LC_50_ value of about 5 mg/L indicates that CuFeO_1A is significantly less toxic than CuZnFeO_2A, whose LC_50_ was about 0.042 mg/L. This suggests that CuFeO_1A may be safer for the environment, but it still has moderate toxicity and should be handled with care.

**Figure 10:**
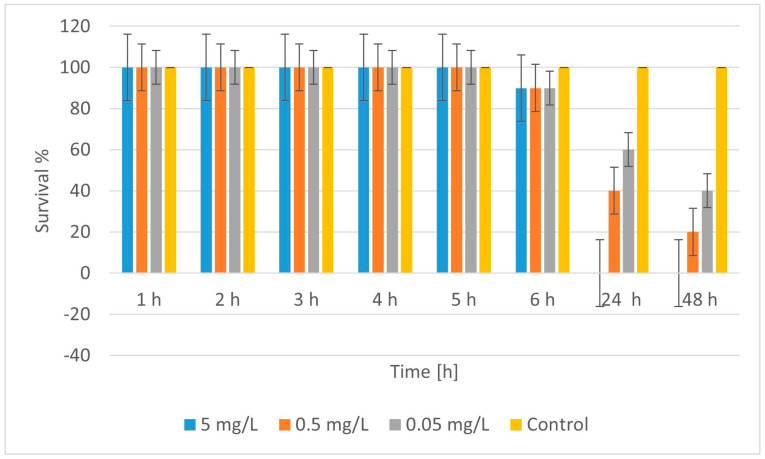
Survival of *Daphnia magna* treated with CuZnFeO_2A.

Figure 10 reflects the results of the treatment of *D. magna* with CuZnFeO_2A, showing that at a high concentration (5 mg/L), 90% of daphnia remained alive during the first 6 h. After 24 h, survival dropped sharply to 0% and did not recover after 48 h. This indicates a strong toxicity of this concentration, causing mortality almost entirely after the first day. At an average concentration (0.5 mg/L) during the first 6 h, survival was also high (90%). After 24 h, survival dropped to 40%, and at the 48^th^ h to 20%. The toxic effect occurred more slowly and was not completely lethal at these concentrations, but it was significant. At a low concentration (0.05 mg/L), survival during the first 6 h was 90%. After 24 h, it dropped to 60%, and at 48 h, to 40%. There was still a significant toxicity, but the effect was weaker compared to higher concentrations. Therefore, CuZnFeO_2A had a toxic effect on daphnia, which was mainly manifested after the first 6 h of exposure. Toxicity was concentration-dependent; higher concentrations cause faster and greater mortality. At the 48^th^ h, mortality ranged from 60% at 0.05 mg/L to 100% at 5 mg/L.

The toxic effect of CuZnFeO_2A was not instantaneous but developed over time, probably due to accumulation or a delayed cellular/molecular response. The toxic effect was strongly concentration-dependent. An LC_50_ of ~0.0417 mg/L was calculated that indicates that even low concentrations of CuZnFeO_2A are toxic to daphnia. The control group (without nanoparticles) showed 100% survival at all times, confirming that mortality was due to CuZnFeO_2A exposure. The ecological significance is related to the fact that daphnia is a key component of freshwater ecosystems, and the toxicity of CuZnFeO_2A, even at low concentrations, can have significant consequences for the ecological balance. This requires careful monitoring and assessment of the possible use or disposal of these nanomaterials in the environment.

**Figure 11:**
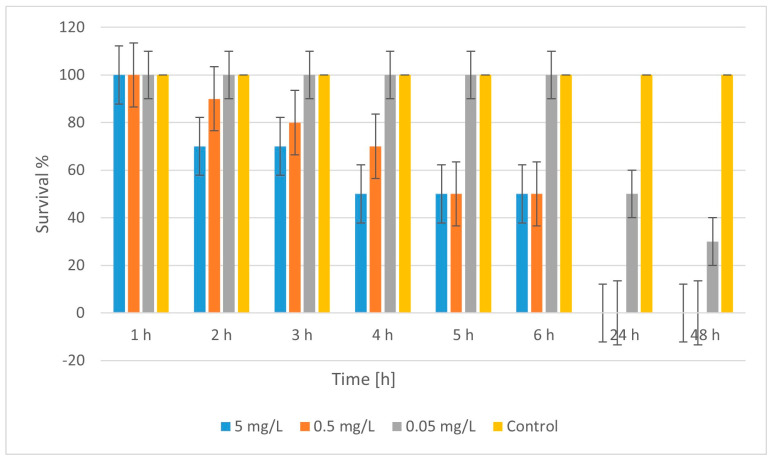
Survival of *Daphnia magna* treated with CuFeO_3A.

In Figure 11, the results of the time dynamics of CuFeO_3A toxicity showed that at 5 mg/L, survival dropped in the first few hours, from 100% to 70%, after 2 h, and reached 50% at 4–6^th^ h, after which by the 24^th^ and 48^th^ h, survival was nil.

At 0.5 mg/L, there was also a visible decrease in the first 6 h, with 50% survival at 5–6^th^ h, and complete mortality at the 24^th^ and the 48^th^ h. At 0.05 mg/L, there was no mortality for up to 6 h, but after 24 h, survival dropped to 50%, and at the 48^th^ h, to 30%, which indicates a delayed toxic effect at the low concentration. The control was stable with 100% survival. Regarding the concentration and time-dependent effect, it is clear that the toxicity of CuFeO_3A is influenced by the concentration and time. The higher the concentration, the faster and more severe mortality occurs. At low concentrations (0.05 mg/L), the toxic effect was delayed and was smaller, but it was still significant after 24–48 h. The LC50 (for 48 h) was calculated by linear interpolation for LC_50_ (between 0.05 and control). LC_50_ (48 h) ≈ 0.036 mg/L CuFeO_3A. It is evident that CuFeO_3A is highly toxic to daphnia, with its toxicity being similar to that of CuZnFeO_2A (LC_50_ around 0.04 mg/L). Toxicity was also manifested as an acute effect at higher concentrations (mortality within 6 h) and more delayed at low concentrations. The control confirmed that mortality is due to exposure to CuFeO_3A.

**Figure 12:**
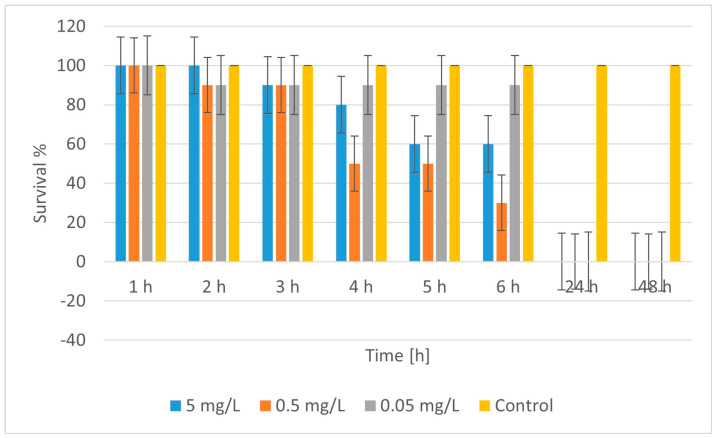
Survival of *Daphnia magna* treated with CuFeO_4A.

The results regarding the time dynamics of CuFeO_4A toxicity, Figure 12, showed that at 5 mg/L and 0.5 mg/L, there was a gradual decrease in survival within the first 6 h. At 5 mg/L, survival dropped from 100% to 60% in 5–6 h. At 0.5 mg/L, the drop was even more drastic, at 30% at the 6^th^ h. After 24^th^ and 48^th^ h, at all concentrations, survival was 0%, i.e., complete mortality. The concentration-dependent effect showed that higher concentrations lead to faster and stronger toxicity. Even the lowest concentration (0.05 mg/L) caused complete mortality within 24 h, although during the first 6 h, survival was about 90%. Since mortality was 100% at all concentrations tested at the 24^th^ and 48^th^ h, it was not possible to calculate LC_50_ from these data, because even at the lowest concentration, mortality was complete. Therefore, CuFeO_4A is very toxic to daphnia, even at the lowest test concentration (0.05 mg/L), resulting in 100% mortality within 24 h. Toxicity began within the first few hours, but the effect was delayed, with a gradual decrease in survival. For a more accurate determination of LC_50_, it is necessary to study lower concentrations (below 0.05 mg/L). The control test confirmed that the mortality was caused by the exposure to the presumed active substance.

**Figure 13:**
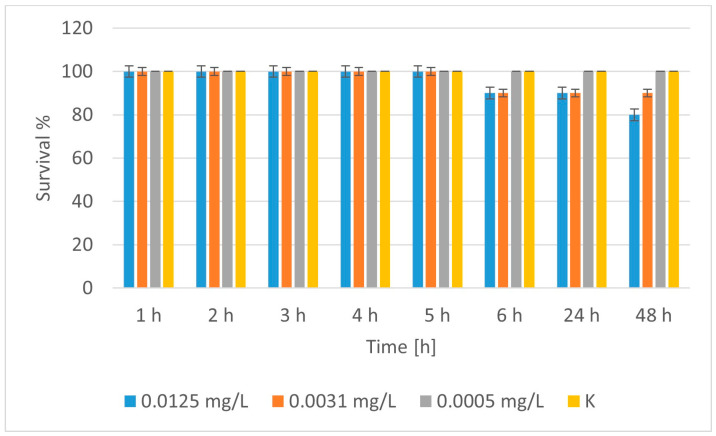
Survival of *Daphnia magna* treated with nCuO.

The treatment of *D. magna* with nCuO showed 100% survival of daphnia in the control group at all time points, Figure 13, confirming that there were no external factors affecting mortality. At the lowest concentration (0.0005 mg/L), there was no observed effect on survival. All daphnia survived (100%) for 48 h. This is probably the NOEC (No Observed Effect Concentration). At a concentration of 0.0031 mg/L, there was no effect until the 5^th^ h, but from the 6^th^ h onwards, 10% mortality (90% survival) was observed. There was no further deterioration until the 48^th^ h. This concentration can be defined as the LOEC (Lowest Observed Effect Concentration). At the highest concentration (0.0125 mg/L), 100% survival was observed by the 5^th^ h, 90% by the 6^th^ h, and a decrease to 80% by the 48^th^ h. It is clear that the exposure time enhances the toxic effect of the nanoparticles. Therefore, time- and dose-dependent toxicity of nCuO nanoparticles to daphnia was observed. The higher the concentration and the longer the exposure, the greater the mortality. The concentration of 0.0005 mg/L can be considered safe (NOEC) for the period of 48 h. The concentration of 0.0031 mg/L showed the onset of toxic effects (LOEC), and 0.0125 mg/L already led to significant mortality with prolonged exposure. We did not access concentrations at which the mortality was above 50%, i.e., there was no clear zone around LC_50_. The highest mortality we observed was 20% (at 0.0125 mg/L). Therefore, LC_50_ > 0.0125 mg/L, but we cannot provide an accurate estimate at this point. This substance can be classified as nontoxic or weakly toxic to daphnia. The toxicities of all samples are summarized in Table 5.

OECD/REACH toxicity classes (a simplified scheme for categorizing the toxicity of substances based on LC_50_):

- LC_50_ ≤ 1 mg/L → Very toxic;
- 1 < LC_50_ ≤ 10 mg/L → Toxic;
- LC_50_ > 10 mg/L → Slightly toxic;
- LC_50_ not reached → Slightly or nontoxic.

The ultimate recipient of nanoparticles, including various types of copper-based nanoparticles, is the aquatic environment [84]. These recent results on nanoparticle ecotoxicity, with respect to an anticipated increased production, especially as a component of antimicrobial agents, raise concerns about their uncontrolled release into the environment and the subsequent ecological risks. The high reactivity of Cu-based particles allows for interactions with biotic and abiotic components of the environment, leading to bioaccumulation and disruption in living organisms. Increasing the concentration of Cu-based particles causes various toxic effects, mainly by inducing oxidative stress, disrupting antioxidant mechanisms, damaging cells and tissues, and slowing growth. The need for further research on their toxicity and measures to control their release into the environment is emphasized. Given the limited data on the toxicity of copper-based nanoparticles, additional studies are needed. Some earlier studies [85] found that low exposure concentrations of Cu nanoparticles and ZnO nanoparticles (below 0.05 and 0.5 mg/L, respectively) accumulated mainly dissolved ions, while at high exposure concentrations (above 0.1 mg/L and 1 mg/L, respectively), particles rather than released ions played a dominant role in the accumulation process. The toxicity and accumulation of Cu as well as ZnO nanoparticles in *Daphnia magna* depended on the particle size, exposure concentration, and routes of uptake by the organism. Metal nanoparticles can inhibit the growth and reproduction of *D. magna*, with effects that differ depending on whether they are particles, dissolved ions, or mixtures. The results of another study [86] showed that nCu was toxic in all endpoints. The 48 h median lethal concentration of nCu was 0.63 mg/L. Significant inhibition of the reproduction and growth of *D. magna* was found. A concentration-dependent decrease in filtration and uptake was observed, which was consistent with the inhibition of the reproduction and growth of *D. magna*. Biochemical response tests showed an increase in glutathione S-transferase activity and a decrease in acetylcholinesterase activity, while superoxide dismutase and catalase activities increased at low concentrations and decreased at high concentrations for all exposures. These results confirm that nanosized Cu and Cr can have negative effects on *Daphnia magna* at various levels [86]. As far as our study is concerned, a special note is left with regard to the CuFeO_3A material, which appears most toxic, while at the same time we did not observe the explicit presence of copper-based phases in the corresponding diffraction pattern, represented in Figure 2. The reason hence, may well be that in that sample non crystalline or very small crystallite size copper and/or copper oxide phases have been formed, which are more toxic than the larger crystallite copper of nCuO.

The accumulation in the antennae of *Daphnia magna* during our tests is additionally illustrated in Figure S2.

## 3. Materials

Fe(Cl)_3_.6H_2_O, CuCl_2_, ZnCl_2_, and NaOH all > 99% purity metals and 25%wt NH_4_OH from AnalytiChem Belgium NV were used along with extra-pure, SLR-grade ethylene glycol from Fisher scientific, and PEG6000, Sigma-Aldrich, St. Louis, MO, USA.

The materials used in the chemiluminescent study were purchased with high purity: iron sulphate (p. a.) (Merck, Darmstadt, Germany), hydrogen peroxide (30%) (Merck, Darmstadt, Germany), phenazine methosulfate (PhMS) (N-methyldibenzopyrazine methyl sulfate salt) (p. a.) (Merck, Germany), lucigenin (bis-N-methylacridinium nitrate) (p. a.) (Sigma-Aldrich, St. Louis, MO, USA), β-nicotinamide adenine dinucleotide, reduced form (p. a.) (NAD.H, Boehringer, Ingelheim am Rhein, Germany), dimethyl sulfoxide (p. a.) (DMSO, Sigma-Aldrich, USA), buffers pH 8.5 and pH 7.4 (Sigma-Aldrich, St. Louis, MO, USA). All chemicals were used as purchased.

## 4. Methods

### 4.1. Nanocrystalline Materials’ Preparation and Characterization

The synthesis procedures for the investigated nanopowders were similar to those reported in Ref. [67], with minor modifications. All details are given in the Supplementary Information accompanying this article.

The phase composition of the synthesized materials was checked by X-Ray Powder Diffraction (XRPD) using an URD-6 diffractometer, Freiberger Präzisionsmechanik GmbH, Rich. Seifert & Co. GmbH & Co. KG, Freiberg, Germany, using a Cu radiation source and a graphite crystal monochromator at the detector side. The X-ray source was operated at 35 kV and 20 mA. Powder diffraction patterns were collected with a 2θ step of 0.05 degrees, and 10 s counting time per step. Coherent scattering length (crystallite size) was estimated from the broadening of the well-separated (333) diffraction line of the spinel phase, using Scherrer’s equation:

(4) $$D_{hkl}=\frac{K\lambda }{\beta cos\theta }$$

in which *K* is Scherrer’s constant (0.9), λ is the radiation wavelength of the copper source, and β is the Full Width at Half Maximum (FWHM) in radians of 2*θ*. Unit cell parameters were determined by Rieteveld refinements of the PXRD data with the aid of the FullProf software suite [87], version 7.5.

Transmission electron microscopy (TEM) was performed on a high-resolution, 200 kV STEM JEOL JEM 2100 instrument with JEOL, Akishima, Japan. TEM images were taken using a CCD camera (GATAN Inc., Pleasanton, CA, USA). Scanning Electron Microscopy (SEM) was performed on a TESCAN Lyra I XMU (Brno-Kohoutovice, Czech Republic) equipped with a Quantax 200 X-ray Energy Dispersive Spectrometer (EDS) from Quantax, Billerica, MA, USA.

Magnetic properties were characterized using a Vibrating Sample Magnetometer (VSM) at room temperature, with maximal magnetic field of 6 kOe. The samples were prepared by compressing the powder into cylindrical quartz containers so that the particles could not move during the measurements.

### 4.2. Chemiluminescent Assay

The first system was Fenton’s. It generated ^·^OOH and ^·^OH radicals: it contained 0.2 mol of sodium hydrogen phosphate buffer, with the chosen pH, Fenton’s reagent: FeSO_4_ (5 × 10^−4^ mol)—H_2_O_2_ (1.5%), lucigenin (10^−4^ mol), and the tested material (1 mg/mL).

The second system contained the strong oxidant hydrogen peroxide (H_2_O_2_): 0.2 mol sodium hydrogen phosphate buffer, with the chosen pH, H_2_O_2_ (1.5%), the chemiluminescent probe lucigenin (10^−4^ mol), and the tested material (1 mg/mL).

The third system was generating superoxide radicals (O_2_^·−^) in the reaction NAD.H-PhMS: it contained 0.2 mol of sodium hydrogen phosphate buffer with the chosen pH, NAD.H (10^−4^ mol), phenazine-metasulfate (10^−6^ mol), lucigenin (10^−4^ mol), and the tested material (1 mg/mL).

The control samples presented blank reactions, where the tested new material was not applied. All reactions were monitored for 3 min, every 3 s, at 37 °C. All materials were sonicated for 60 min before application.

All experiments were performed in triple reproducible measurements. The statistical analysis was performed by Origin 8.5 and Microsoft Office Excel 2010 and Student’s *t*-test, *p* ≤ 0.05, measured by LUMIstar Omega (BMG Labtech GmbH, Ortenberg, Germany, 2020).

### 4.3. Antimicrobial Activity Tests

The antimicrobial effect was tested on Gram-positive and Gram-negative bacteria, *Escherichia coli* ATCC 25922 and *Staphylococcus aureus* ATCC 25923, which were both supplied by the National Bank of Industrial Microorganisms and Cell Cultures (NBIMCC), Sofia, Bulgaria. The test microorganisms were grown in nutrient broth (NB Conda, Madrid, Spain) at 37 °C and 180 rpm for 24 h with 2 subcultivations to reach the exponential growth phase. The microbial density of the cultures was adjusted to 0.5 according to the McFarland standard and used for the antimicrobial tests. Two classical methods were used to study the antibacterial effect of the synthesized nanoparticles: spot and well diffusion tests in agar medium. They were revitalized in nutrient broth (Conda, Spain) and subcultured two times to obtain exponential cultures. The bacterial suspension density was measured and justified to 0.5 according to McFarland on a densitometer, and 100 µL of bacterial suspension was inoculated in 20 mL melted and cooled to 45 °C nutrient agar (Conda, Spain). After solidification of the agar, a bore holder was used to make 6 mm wells in the agar plate, and 50 µL of the nanoparticle dispersions with different concentrations was dropped inside the wells. Petri dishes were kept for 2 h in a refrigerator at 4–5 °C to provide diffusion of nanoparticles in the agar, and after that, they were incubated for 24 h at 37 °C for the development of bacterial cultures. The formed sterile zones around agar wells were measured, and the antibacterial effect was assessed. The spot test was conducted with the same nutrient media and bacteria, but the inoculation of bacterial suspensions was on the solid nutrient agar. Drops of 5 µL of nanoparticle suspension were used, and after 2 h of diffusion in a refrigerator, Petri dishes were incubated for 24 h at 37 °C. The development of bacterial cultures was assessed and compared with the results of the diffusion test. It seems that direct contact between nanoparticles and bacteria produces more precise results than the agar diffusion test.

### 4.4. Environmental Toxicity by Testing the Lethality of Daphnia Magna

Acute ecotoxicity tests were conducted using *Daphnia magna* (Cladocera, Crustacea) for all types of materials, which were applied in three concentrations with three replicates and two controls (untreated daphnia). *Daphnia magna* toxicity test was conducted according to the Acute lethality toxicity protocol OECD (2004) and Guideline for the testing chemicals Acute immobilization test (2012) Test No:202 [88]. *Daphnia* was grown in laboratory conditions. Readings were made at the 1^st^, 2^nd^, 3^rd^, 4^th^, 5^th^, 6^th^, 24^th^ and 48^th^ h. For every experiment, we used 10 daphnia in a glass with water, repeating each experiment three times. A percentage of survival data was handled within Microsoft Excel (LTSC MSO). Average survival percentage and the standard deviation were calculated for every group and time data point.

The mean survival rate (%) and standard deviation were calculated for each group and time point. The results are presented as mean ± standard deviation (SD). For the graphical representation, bar charts were used, with different colors indicating the concentrations, and the errors were visualized using error bars.

## 5. Summary and Conclusions

Our attempts to prepare nanocrystalline copper ferrite via solvothermal syntheses at relatively low temperatures resulted in obtaining composite materials, containing a majority phase of nanocrystalline ferrite spinel, agglomerating around relatively large well-crystallized metallic copper particles. Clearly, at the applied syntheses conditions, Cu(II), being a significantly elongated Jan–Teller ion, does not fit in the otherwise flexible cubic spinel lattice, and the system minimizes its energy by expelling this species. Likely depending on the pH and the oxidizing/reducing nature of the environment, either metallic copper or copper oxides can be formed as more stable secondary phases. When zinc was also added to the starting solution, zinc-substituted ferrite was obtained, yet again nanocrystalline, but notably the phase-separated metallic nanoparticles were also nanocrystalline. All of the freshly prepared materials possess ferrimagnetic or superparamagnetic properties for the Zn-substituted ferrite in particular. The biological activity of the studied nano-ceramics was closely related to their chemical/phase composition as well as the degree of oxidation of the metal cations in their content. The nanomaterials that were dominated by mixed-valence phases showed the strongest and most persistent suppression of the reactions dependent on ROS. The high concentration of Fe^3+^ and the limited presence of reduced Cu^+^ and Fe^2+^ ions diminished the activity of the material and therefore excessive formation of free radicals, maintaining redox balance. On the contrary, phases and nanomaterials that were enriched with reduced metals, e.g., metallic Cu, could be easily oxidized in Fenton’s reaction and exhibit weaker and pH-dependent inhibitory effects. The presence of Zn^2+^ stabilized the crystal lattice; it could reduce the solubility of copper. However, Zn^2+^ itself did not participate in redox cycles and could weaken the antioxidant potential by diluting the active iron and copper centers. The pure nCuO sample demonstrated the highest antibacterial activity, because of typical pronounced oxidative and toxic effects. The inclusion of Fe^3+^ and Zn^2+^ in the lattice stabilized the copper species and limited oxidative stress, transforming a potentially prooxidant system into a more biologically compatible redox modulator. Those chemical dependencies probably determined the described inhibitory properties in the redox tests, as well as the weak bactericidal properties for most of the tested nanomaterials. Despite the materials’ complex nature, all materials show very similar antimicrobial properties, being generally nontoxic and slightly more efficient against the Gram-positive *E. coli*. The redox activity tests proved that all sample compositions and related particle morphologies have good physiological compatibility, do not promote ROS accumulation, and maintain appropriate redox balance. These composite nano-ceramics would not burden the physiological processes that are connected with ROS generation; they would inhibit and suppress such reactions. Acute tests with *Daphnia magna*, on the other hand, evidence high ecotoxicity for all of the investigated active materials, likely due to the presence of fresh metallic copper particles. Their high ecotoxicity was probably due mainly to mechanical effects in the tested organisms, possibly facilitated by the magnetic properties of the complex particle aggregates, further accompanied by chemical transformations leading to metabolic damages. These latter results strongly suggest that such materials should be carefully characterized and classified to identify the correct phase compositions, consequently released into the environment or disposed of with care, but could at least be controlled by not very strong external magnetic fields.

## Figures and Tables

**Table 1:** Samples identification and physical parameters: given is the lattice parameter of the conventional cubic Bravais unit cell, representative crystallite size from the Scherrer dependence of the line (333) broadening, the maximum attained magnetic moment, M_max_, at 6000 Oe, and the coercitivities, H_C_.

Sample	Phase	Latt. Par., [Å]	Cry. Size, [nm]	M_max_, [emu/g]	H_C_, Oe
CuFeO_1A	Magnetite + Cu	8.36 (7)	15	56	89
CuZnFeO_2A	Magnetite + Cu	8.428 (5)	9	27	0
CuFeO_3A	Hematite + Magnetite	5.03 (0), 13.76 (4), 8.34 (6)	n.d.	22	16
CuFeO_4A	Magnetite + Cu	8.37 (7)	11	46	0

**Table 2:** Inhibitory and prooxidant effects of nano-ceramic materials in ROS-generating systems.

SYSTEM/ROS	pH, 37 °C	OBSERVATION	MOST ACTIVE MATERIALS	EFFECT (FOLDS)	TYPE OF EFFECT
Fenton’s system (^·^OH, ^·^OOH, H_2_O_2_, ^−^OH)	8.5 (optimal)	all materials showed definitive inhibitory activity	CuFeO_3A CuFeO_4A	128× 113×	strong inhibition
Fenton’s system (^·^OH, ^·^OOH, H_2_O_2_, ^−^OH)	7.4 (physiological)	all materials suppressed oxidation	CuFeO_1A CuZnFeO_2A	4.5× 4.2×	moderate inhibition
H_2_O_2_ system	8.5 (optimal)	all materials inhibited oxidation	CuFeO_4A	88×	strong inhibition
H_2_O_2_ system	7.4 (physiological)	well-distinguished suppression of the oxidation	CuO	~6×	clear inhibition
O_2_^·−^ radicals	8.5 (optimal)	strong inhibition observed	CuFeO_1A	8.8×	strong inhibition
O_2_^·−^ radicals	7.4 (physiological)	oxidation suppression converted into prooxidant effect; only CuFeO_4A showed inhibition	CuFeO_1A (prooxidant), CuFeO_4A (inhibitory)	CuFeO_1A > 2.4× CuFeO_4A < 2×	prooxidant/weak inhibition

**Table 3:** Agar well test results against *Escherichia coli* at various nanomaterial concentrations, 10.0, 5.0, 2.5, 1.0, and 0.1 mg/mL, including a nanocrystalline sample of CuO as a reference.

Active Material	10 mg/mL	5 mg/mL	1.0 mg/mL	0.1 mg/mL
nCuO	BC	BC	MBC	MIC
CuFeO_1A	MBC	MIC	No Effect	No Effect
CuZnFeO_2A	MBC	MIC	No Effect	No Effect
CuFeO_3A	MBC	MIC	No Effect	No Effect
CuFeO_4A	MBC	MIC	No Effect	No Effect

**Table 4:** Agar well test results against *Staphylococcus aureus* at various concentrations of the powder material: 10.0, 5.0, 2.5, 1.0, and 0.1 mg/mL, for all tested materials as well as the nanocrystalline nCuO reference material.

Active Material	10 mg/mL	5 mg/mL	1.0 mg/mL	0.1 mg/mL
nCuO	BC	BC	MBC	MIC
CuFeO_1A	No Effect	No Effect	No Effect	No Effect
CuZnFeO_2A	MBC	MIC	No Effect	No Effect
CuFeO_3A	No Effect	No Effect	No Effect	No Effect
CuFeO_4A	MBC	MIC	No Effect	No Effect

**Table 5:** Summary of the LC_50_ (48 h) for the tested substances on *Daphnia magna*.

No.	Material	LC_50_ (48 h) [mg/L]	Toxicity Class *
1	CuFeO_1A	~5.0	Moderately toxic
2	CuZnFeO_2A	~0.042	Highly toxic
2	CuFeO_3A	~0.036	Highly toxic
5	CuFeO_4A	<0.05 (100% lethality)	Highly toxic
6	nCuO	>0.0125 (n.d. LC_50_)	Nontoxic/weakly toxic

* Substance LC_50_ (48 h) [mg/L].

## Data Availability

The raw data supporting the conclusions of this article will be made available by the authors on request.

## Supplementary Materials

The following supporting information can be downloaded at: https://www.mdpi.com/article/10.3390/molecules30224454/s1, Samples synthesis details, EDS analyses and element distribution mapping, Figure S1: PXRD patterns of the Cu-containing ferrites around the copper metal (111) and (200) reflexes in the left block. The complex line near 43 degrees, composed by the magnetite (400) and copper (111) lines for the CuZnFeO_2A sample, is shown in the right block. Figure S2: Rietveld fit, Fullprof Ver. 7.5 [S1], of the CuZnFeO_2A PXRD data. Figure S3: PXRD pattern of the nanocrystalline CuO, tenorit, reference material. Figure S4. Deposits from the nanoparticle material on the antennae of *Daphnia magna*—orange deposits on the antennae of daphnia may result from exposure to metal nanoparticles—binding of Cu to surface proteins of the antennae or precipitation of copper compounds.
