# Supplementary material for: Structure, Ecotoxicity, Redox and Bactericidal Activity of Cu-Containing Nanocrystalline Ferrites
Source: Molecules. 2025 Nov 19;30(22):4454. doi: 10.3390/molecules30224454 (PMC12655667; doi:10.3390/molecules30224454)
Supplement: Supplementary file 1 [file molecules-30-04454-s001.zip › molecules-3952404-supplementary.pdf]

Supplementary Information for:

Structure, Ecotoxicity, Redox and Bactericidal Activity of Cu-Containing Nanocrystalline Ferrites

Todor R. Karadimov[1], Elena P. Nenova[2], Elitsa Pavlova[3], I. Ivanova[2], Milena T. Georgieva[1], Peter A. Georgiev[1*]

[1]Faculty of Physics, Sofia University “St. Kliment Ohridski”, Department of Condensed Matter Physics and MicroelectronicS, Sofia, 1164, Bulgaria;

[2]Faculty of Biology, Sofia University “St. Kliment Ohridski”, 8 Dragan Tsankov Blvd, 1164 Sofia, Bulgaria;

[3]Faculty of Physics, Sofia University “St. Kliment OhridskiOptics and Spectroscopy Department, Sofia, 1164, Bulgaria;

Samples preparation

Fe(Cl)3.6H2O, CuCl2, ZnCl2 and NaOH all >99% purity metals basis and 25%wt NH4OH from AnalytiChem Belgium NV were used along with extra pure, SLR grade Ethylene glycol from Fisher scientific, and PEG6000, Sigma Aldrich.

CuFeO_1A 10 mmol $CuCl_2$ and 20 mmol $Fe(Cl)_3.6H_2O$ were dissolved in 25 ml of Ethylene glycol in a glass vial, on a magnetic stirrer at 50 ^oC until clear solution was obtained. Then 2.4 g NaOH dissolved in 4 ml deionized water were dropwise added to the above solution while mixing. The pH of the resultant solution was further adjusted to about 10 by dropwise adding 25% ammonia water solution. At this point 1g of PEG6000 was added. The mixture was further stirred at 80 ^oC for 40 min, then transferred to a 100 ml Teflon lined autoclave and heated to 200 ^oC for 3 days in a convection oven. The reactor was allowed to cool down to room temperature, and the sample was then removed with the help of a neodymium magnet. The powder was then three times washed by redispersing in 50 ml DI water and lastly in 40 ml MeOH. Finally, the collected powder was dried overnight in the oven at 80 ^oC, in air.

CuZnFeO_2A 2 mmol $CuCl_2$, 2 mmol $ZnCl_2$ and 8 mmol $Fe(Cl)_3.6H_2O$ were grinded by hand in a pestle till visibly homogeneous mixture was obtained. Then 1.6 g of NaOH was added and the mixture was grinded again to obtain homogeneous paste. Then 40 ml DI water was added and stirred again. The so obtained solution was transferred in a glass cup and stirred magnetically on a hot plate at 90 ^oC solution temperature, for 90 minutes. A second portion f 30 ml DI water was added and the stirring was continued for about 10 min. Then the solution was allowed to cool down and precipitate. This was filtered out and redispersed in 25 ml ethylene glycol by stirring again at 80 ^oC for about 10 minutes. The mixture was then transferred to 100 ml Teflon lined autoclave thermalized at 180 ^oC, for 24 h. The final pH of the mother liquor at room temperature was 9. Similar washing procedure was applied as above. After draining out the final MeOH, this material was dried at 90 ^oC in a vacuum oven for 2 h.

CuFeO_3A 10 mmol $CuCl_2$ and 20 mmol $Fe(Cl)_3.6H_2O$ were dissolved in 20 ml ethylene glycol by magnetic stirrer, at 60 °C, for 4 h. Then 25% ammonia solution was dropwise added until the pH of the warm solution reached 10-11, (about 10 ml NH_4OH of the solution were necessary) The stirring was continued for additional 2 h, until most of the ammonia evaporated. The mixture was transferred to a 100 ml Teflon lined autoclave at kept at 180 °C, for 16 h. The precipitated powder was extracted and washed as above, then vacuum dried at 90 °C, for 2 h.

CuFeO_4A 3 mmol $CuCl_2$ and 7 mmol $Fe(Cl)_3.6H_2O$ were grinded by hand in a pestle till visibly homogeneous mixture was obtained. Then 1.2 g of NaOH was added and the mixture was grinded again to obtain homogeneous paste. Then 40 ml DI water was added and stirred again. The so obtained solution was transferred in a glass cup and stirred magnetically on a hot plate at 90 °C solution temperature, for 90 minutes. A second portion f 30 ml DI water was added the stirring was continued for about 10 min. Then the solution was allowed to cool down and precipitate. This was filtered out and redispersed in 25 ml ethylene glycol by stirring again at 80 °C for about 10 minutes. The mixture was then transferred to 100 ml Teflon lined autoclave thermalized at 180 °C, for 24 h. After the above-described sample washing procedures, the powder was vacuum dried at 90 °C, for 2 h.

Additional PXRD data

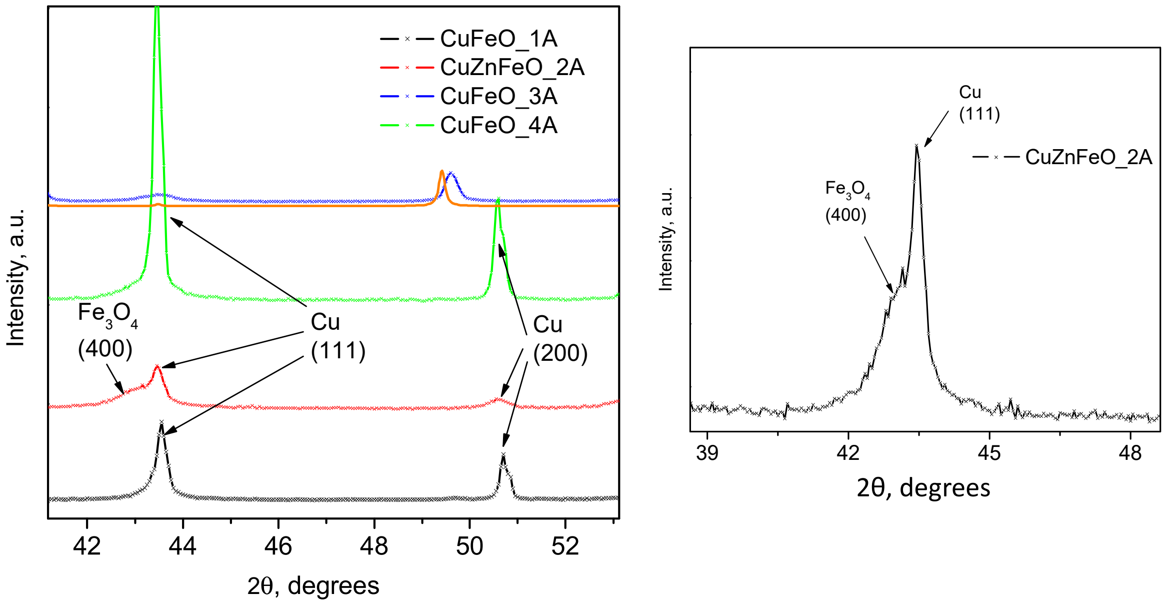

Figure S1. PXRD patterns of the Cu-containing ferrites around the copper metal (111) and (200) reflexes in the left block. The complex line near 43 degrees, composed by the magnetite (400) and copper (111) lines for the CuZnFeO_2A sample is shown in the right block.

Scanning Electron Microscopy (SEM)

CuFeO_1A

Application Note

Sofia University, Faculty of Physics

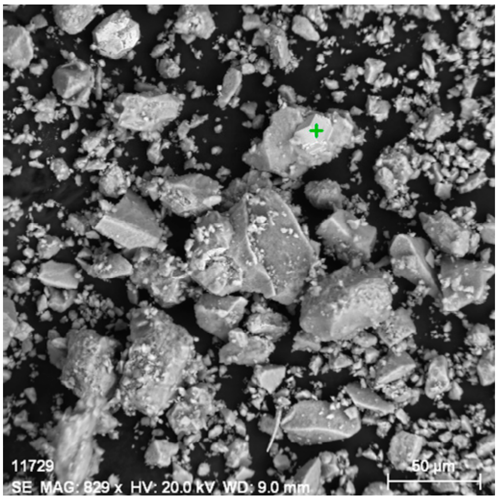

11729Date:7/1/2025 4:05:11 PMImage size:512 x 512Mag:829.372739xHV:20.0kV

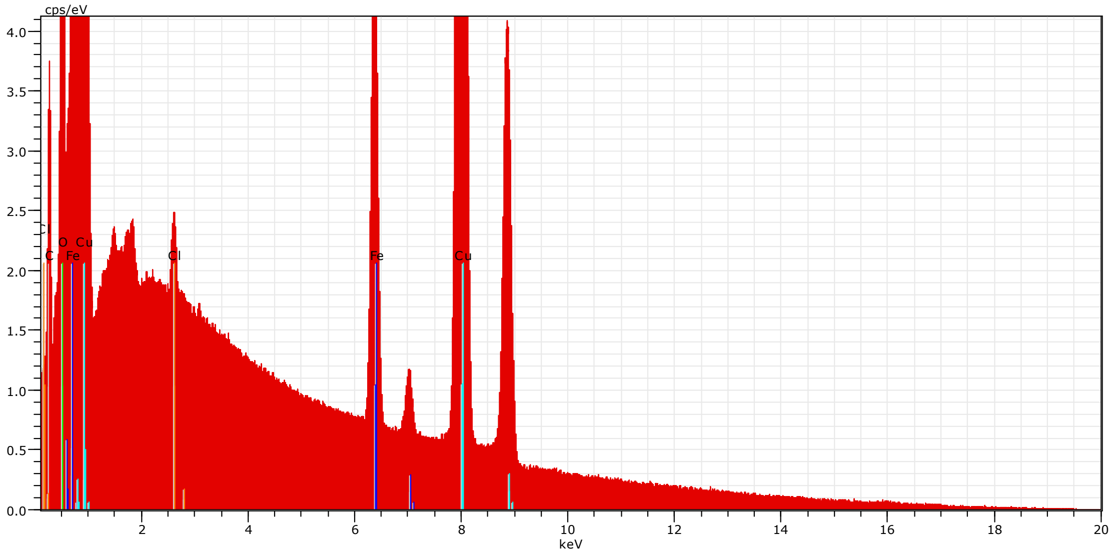

Acquisition 12699 Date:7/1/2025 3:57:16 PM HV:20.0kV Puls th.:36.57kcps

El	AN	Series	unn. C [wt.%]	norm. C [wt.%]	Atom. C [at.%]	Error [%]
C	6	K-series	2.84	3.19	12.13	0.4
O	8	K-series	7.28	8.17	23.32	0.8
Cl	17	K-series	0.40	0.45	0.58	0.0
Fe	26	K-series	5.72	6.43	5.26	0.2
Cu	29	K-series	72.78	81.75	58.72	2.0
		Total:	89.02	100.00	100.00	

Spectrum: Acquisition 12699

El	AN	Series	unn. C [wt.%]	norm. C [wt.%]	Atom. C [at.%]	Error [%]
C	6	K-series	2.84	3.19	12.13	0.4
O	8	K-series	7.28	8.17	23.32	0.8
Cl	17	K-series	0.40	0.45	0.58	0.0
Fe	26	K-series	5.72	6.43	5.26	0.2
Cu	29	K-series	72.78	81.75	58.72	2.0
		Total:	89.02	100.00	100.00	

Application Note

Company / Department

CuFeO_1A

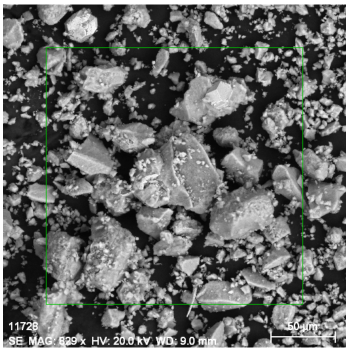

11728Date:7/1/2025 3:54:31 PMImage size:512 x 512Mag:829.372739xHV:20.0kV

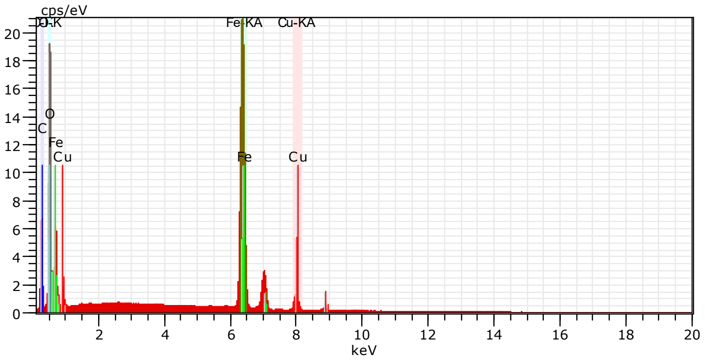

Map Date:7/1/2025 3:43:57 PM HV:20.0kV Puls th.:15.51kcps

Map data 4701Date:7/1/2025 3:43:59 PMImage size:384 x 384Mag:829.372739xHV:20.0kV

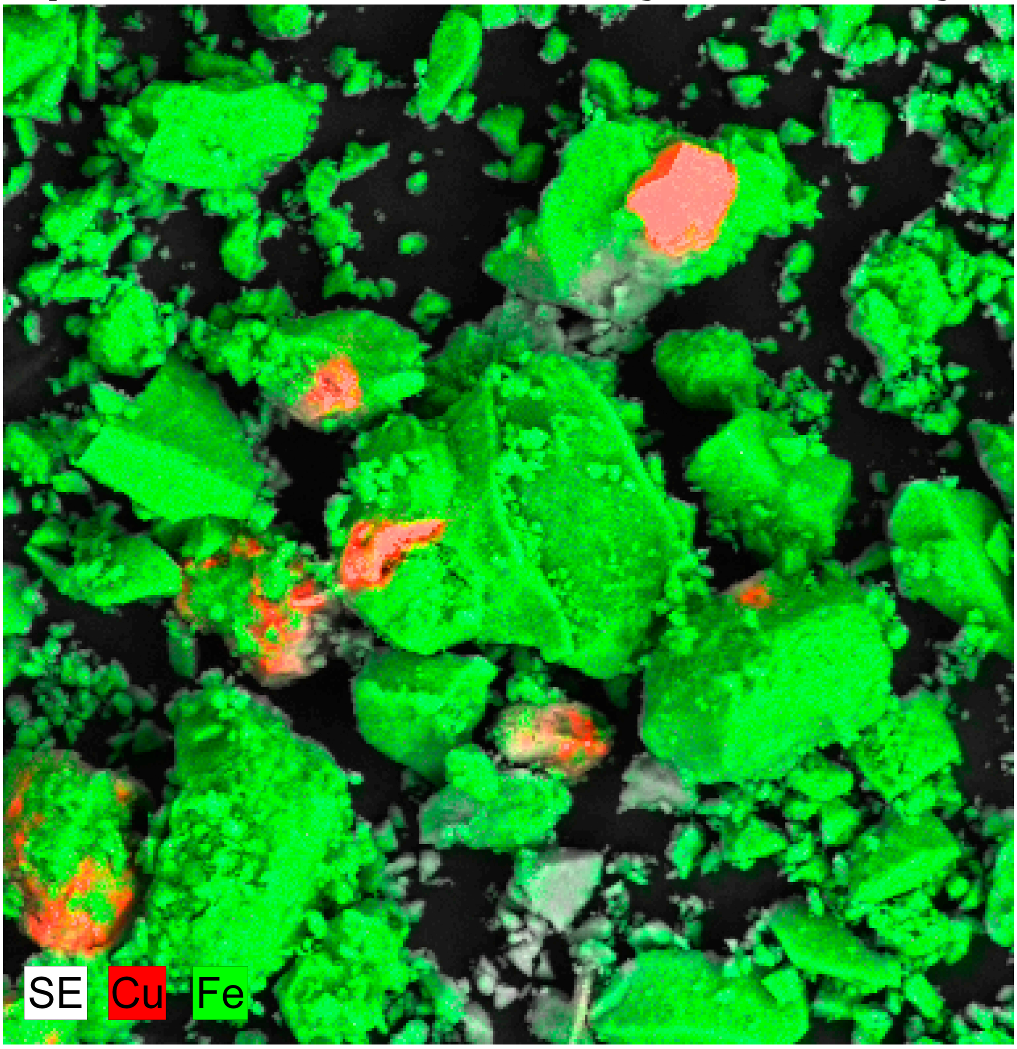

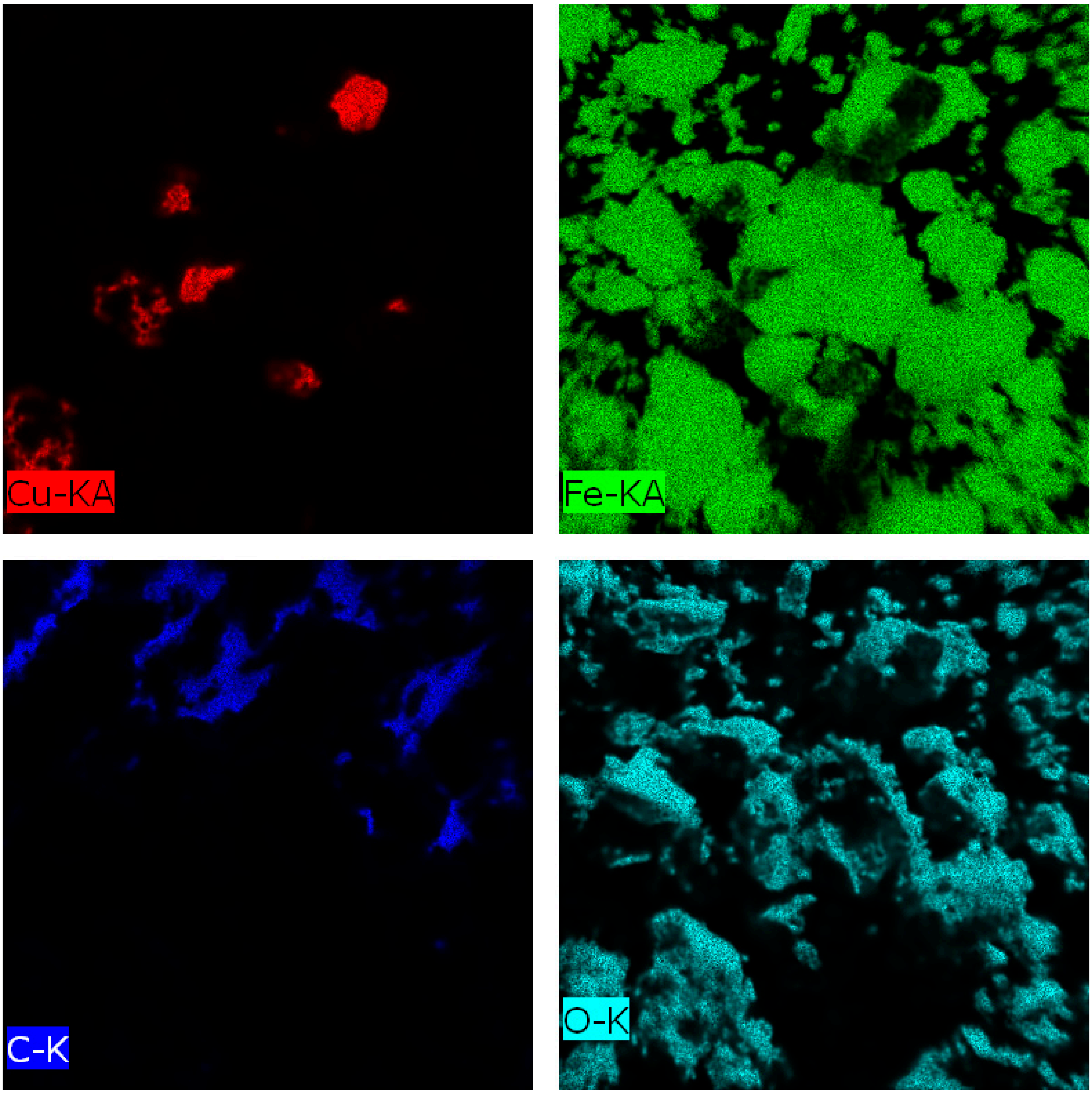
Cu-KA
Fe-KA
C-K
O-K

CuFeO_3A

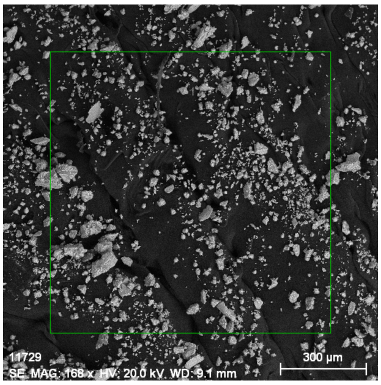

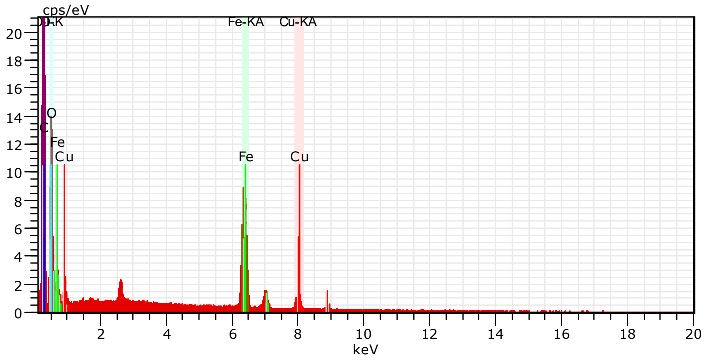

11729Date:7/1/2025 4:20:38 PMImage size:512 x 512Mag:168.306165xHV:20.0kV

Map Date:7/1/2025 4:20:10 PM HV:20.0kV
Puls th.:12.24kcps

Map data 4703Date:7/1/2025 4:20:12 PMImage size:384 x 384Mag:500xHV:20.0kV

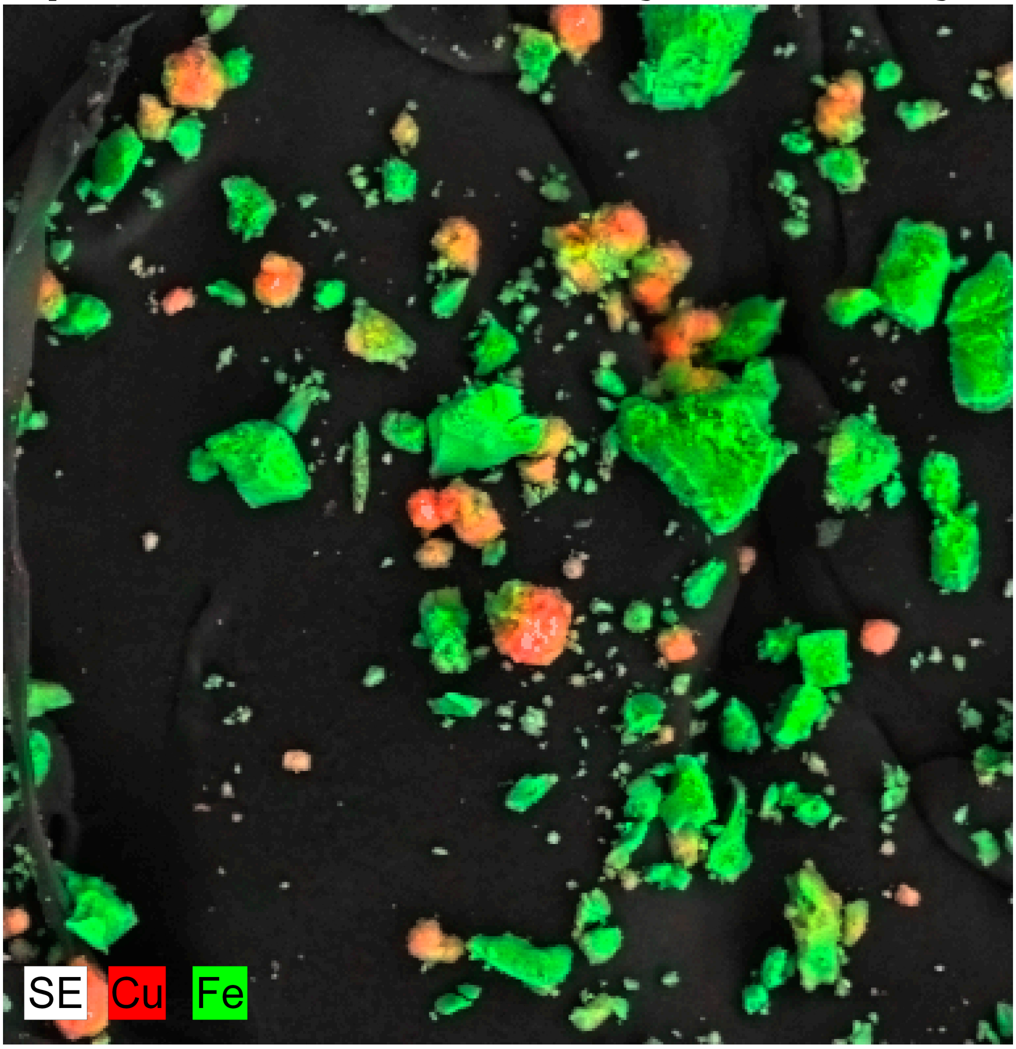

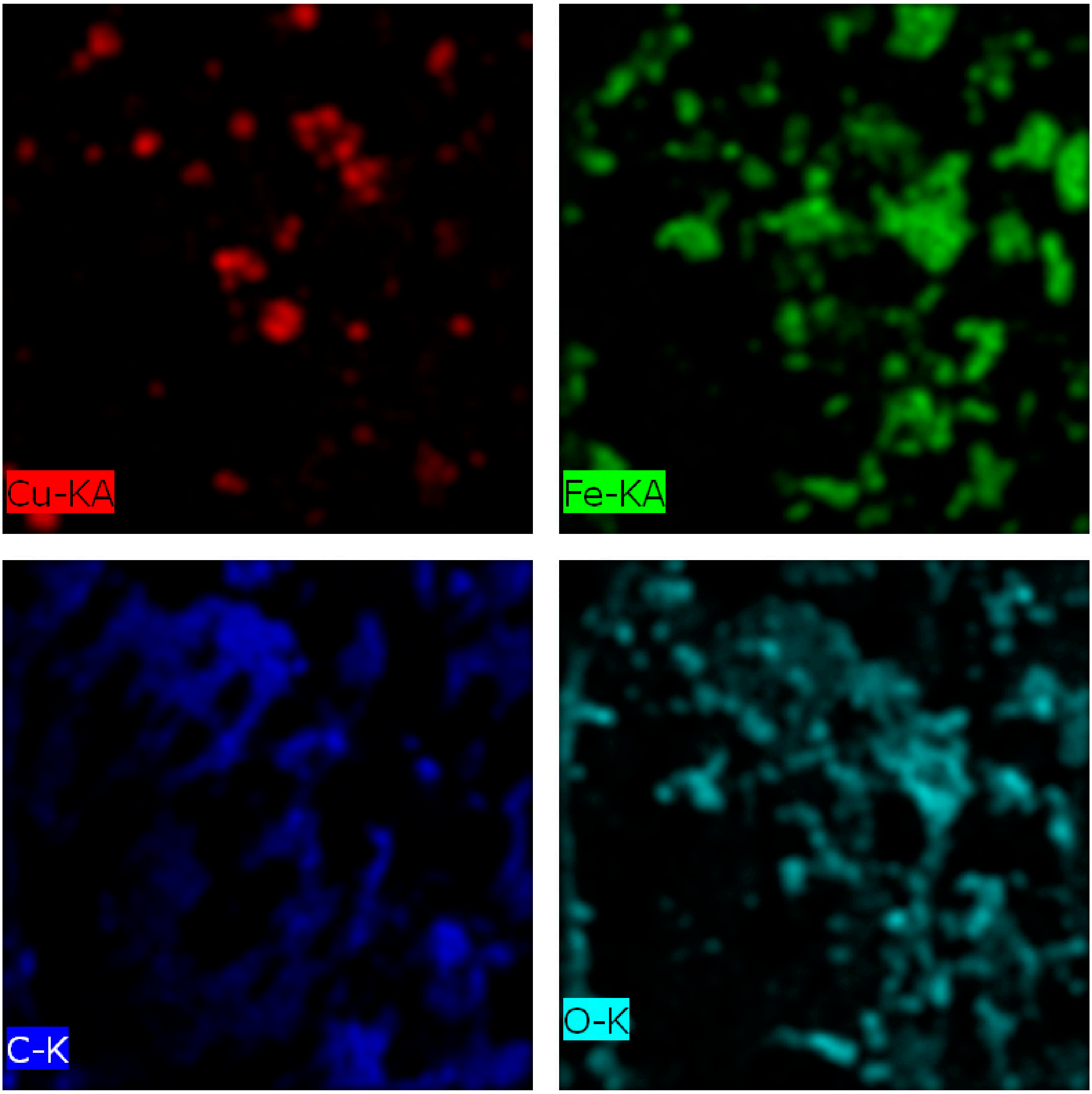

Cu-KA, Fe-KA, C-K, O-KDate:7/1/2025 4:20:38 PM

Image size:384 x 384

Mag:168.306165x

HV:20.0kV

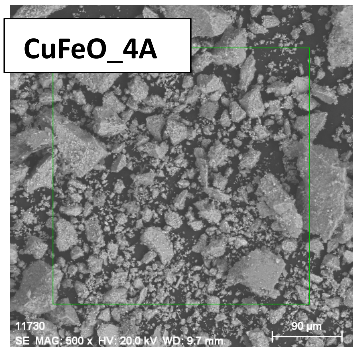

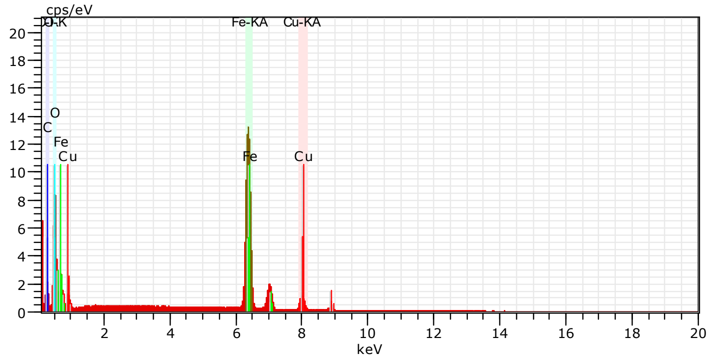

11730Date:7/1/2025 4:37:00 PMImage size:512 x 512Mag:500xHV:20.0kV

Map Date:7/1/2025 4:29:50 PM HV:20.0kV Puls th.:6.93kcps

Map data 4704Date:7/1/2025 4:29:52 PMImage size:384 x 384Mag:500xHV:20.0kV

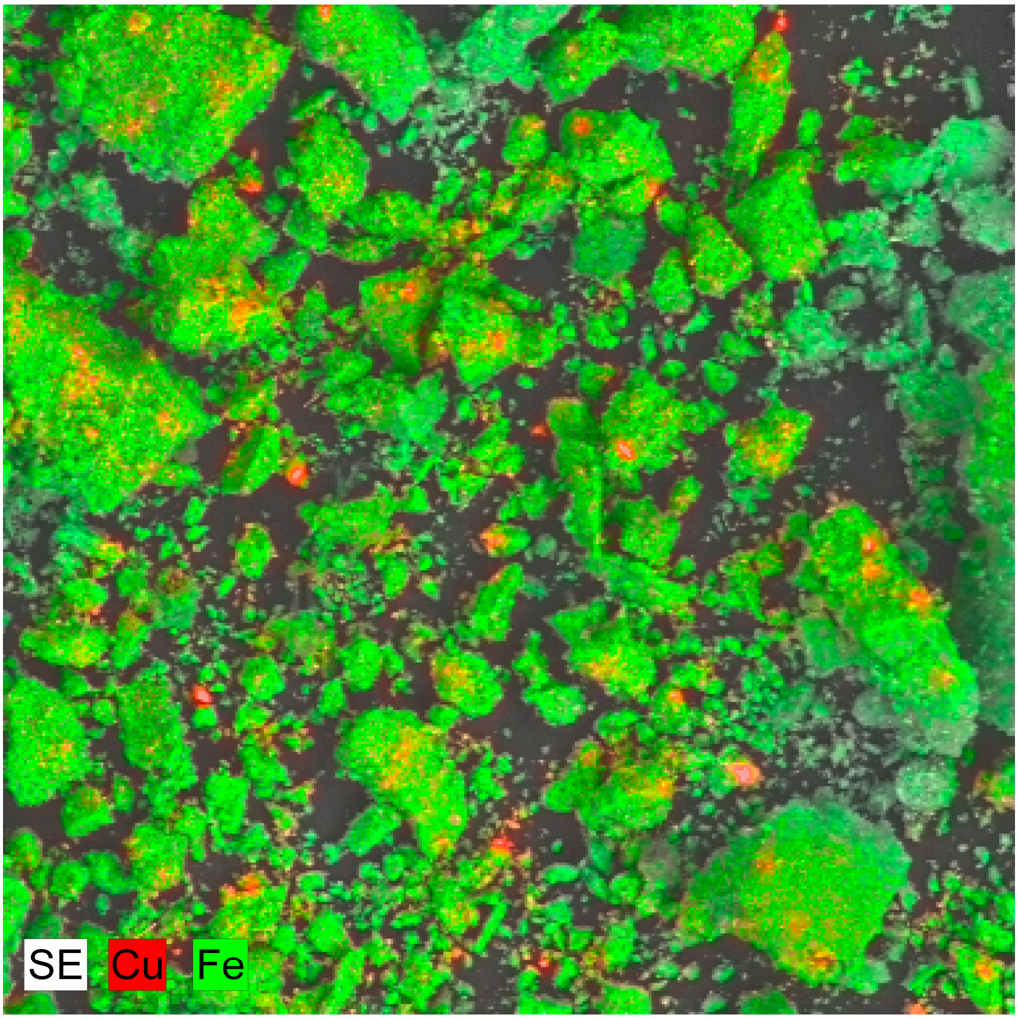

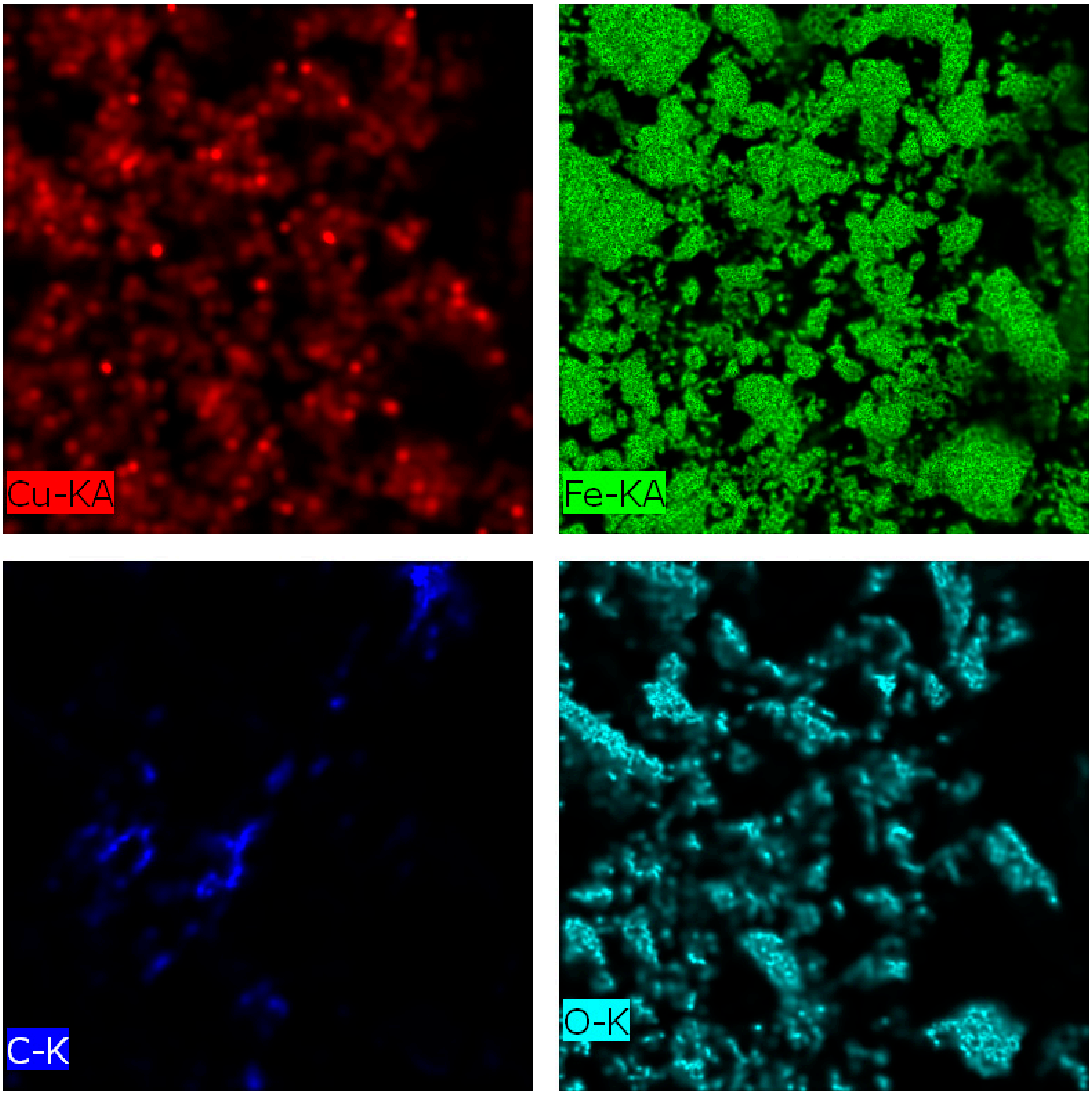

Cu-KA, Fe-KA, C-K, O-KDate:7/1/2025 4:37:10 PM

Image size:384 x 384

Mag:168.306165x

HV:20.0kV

CuO, Tenorit, reference sample TEM results:

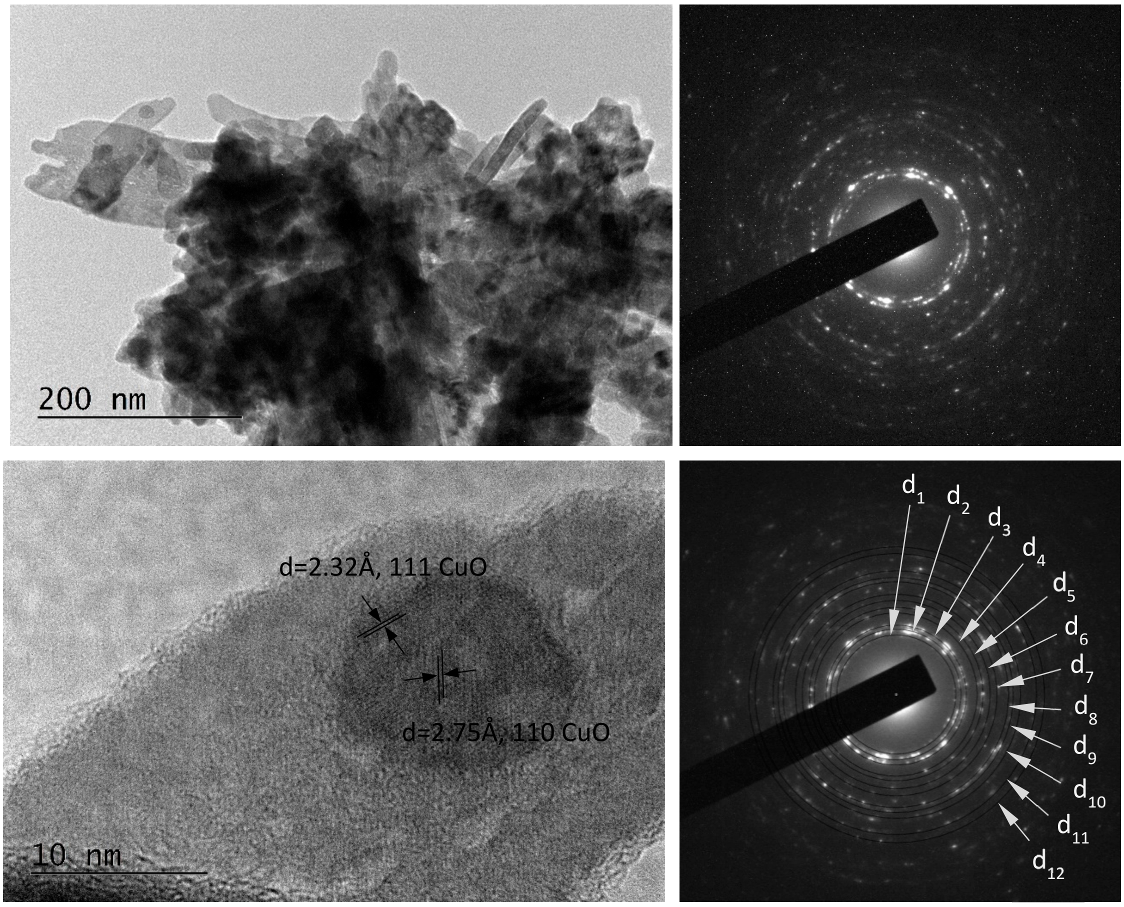

symbol	d [Å]	hkl, phase
d1	2.75	1 1 0
d2	2.52	0 0 2, -1 1 1
d3	2.31	2 0 0
d4	1.96	-1 1 2
d5	1.87	2 0 -2
d6	1.71	0 2 0
d7	1.62	0 2 1
d8	1.42	0 2 2
d9	1.38	1 1 3
d10	1.30	3 1 1
d11	1.15	4 0 0
d12	1.09	-1 3 1

CuO single phase, monoclinic,

a= 4.68330 Å   b= 3.42080 Å c= 5.12940 Å

α= 99.57°, # 96-901-6106;

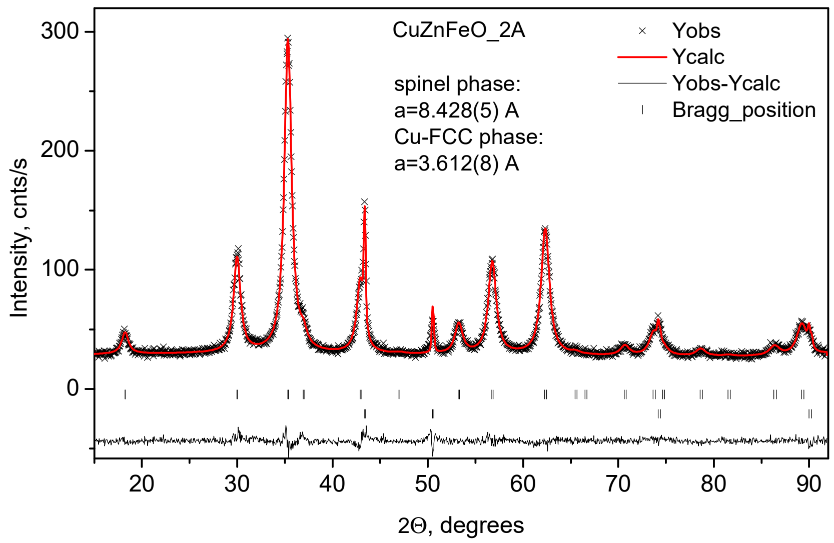

Figure S2. Rietveld fit, Fullprof Ver. 7.5 [87], of the CuZnFeO_2A PXRD data.

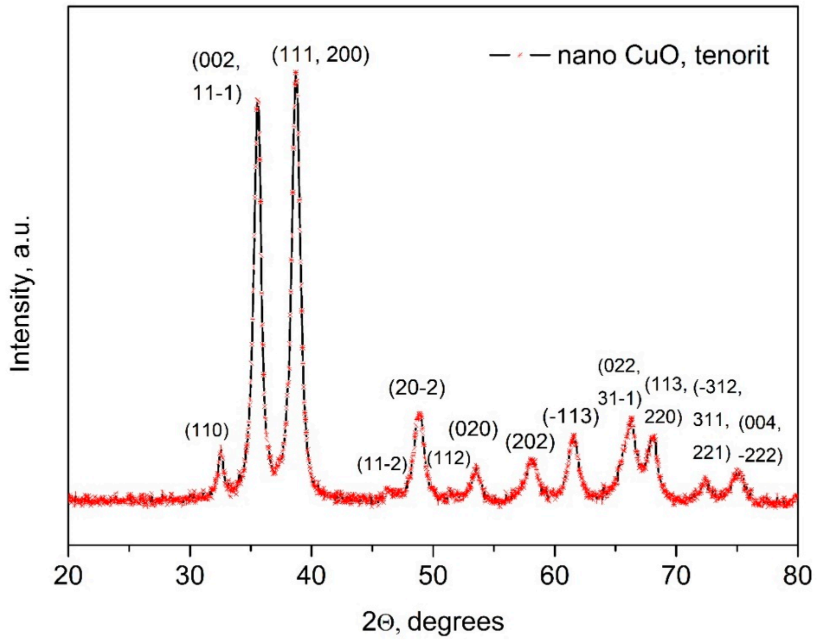

Figure S3. PXRD pattern of the nanocrystalline CuO, tenorit, reference material.

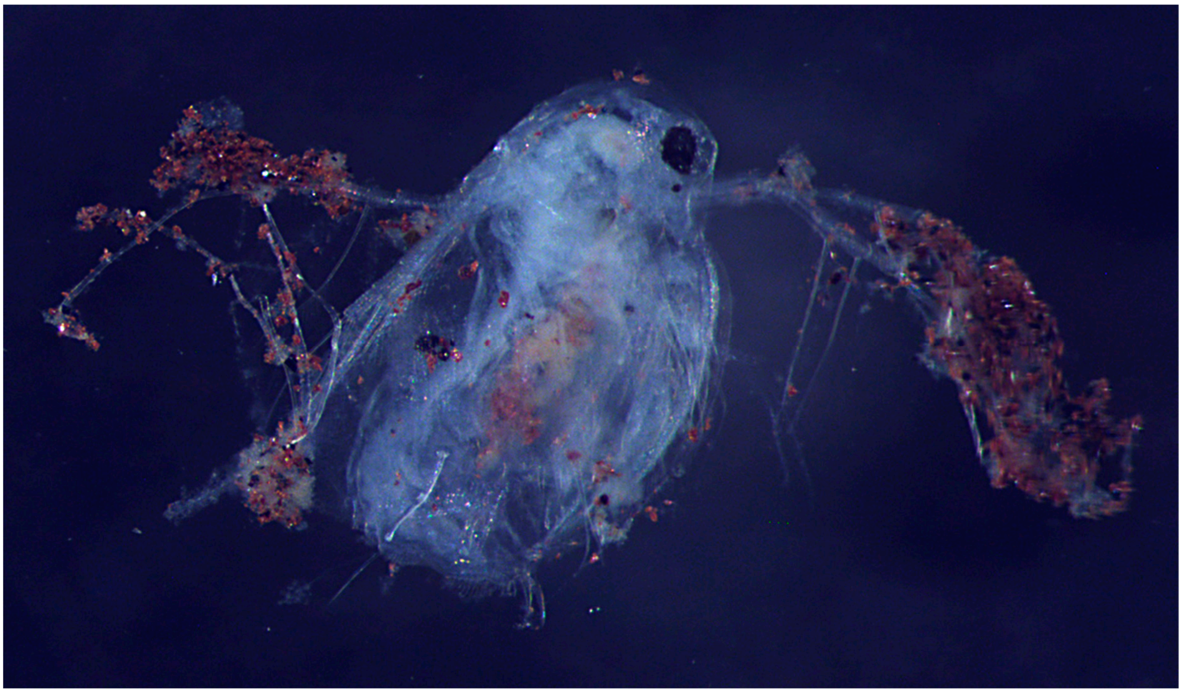

Figure S4. Deposits from the nanoparticle material on the antennae of *Daphnia magna* – orange deposits on the antennae of daphnia may result from exposure to metal nanoparticles - binding of Cu to surface proteins of the antennae or precipitation of copper compounds.

References

87. J. Rodríguez-Carvajal**,** Recent advances in magnetic structure determination by neutron powder diffraction, Physica B 192 (1993) 192 55-69.
